# Comparative effectiveness of common treatments for new-onset atrial fibrillation within the ICU: Accounting for physiological status

**DOI:** 10.1016/j.jcrc.2021.11.005

**Published:** 2022-02

**Authors:** Jonathan P. Bedford, Alistair Johnson, Oliver Redfern, Stephen Gerry, James Doidge, David Harrison, Kim Rajappan, Kathryn Rowan, J. Duncan Young, Paul Mouncey, Peter J. Watkinson

**Affiliations:** a Nuffield Department of Clinical Neurosciences, University of Oxford, Oxford, UK; b Centre for Statistics in Medicine, Nuffield Department of Orthopaedics, Rheumatology & Musculoskeletal Sciences, University of Oxford, Oxford, UK; c Intensive Care National Audit and Research Centre (ICNARC), Holborn, London, UK; d NIHR Biomedical Research Centre, Oxford, UK; e Glowyr ltd., Hawkstone House, Valley Road, Hebden Bridge, West Yorkshire, UK; f Oxford University Hospitals NHS Foundation Trust, UK

**Keywords:** Intensive care, Critical illness, Arrhythmia, Atrial fibrillation, Management, AF, Atrial fibrillation, NOAF, New-onset atrial fibrillation, ICU, Intensive care unit, HR, Hazard ratio, aHR, Adjusted hazard ratio, USA, United States of America, UK, United Kingdom

## Abstract

**Background:**

New-onset atrial fibrillation (NOAF) is common in patients on an intensive care unit (ICU). Evidence guiding treatments is limited, though recent reports suggest beta blocker (BB) therapy is associated with reduced mortality.

**Methods:**

We conducted a multicentre cohort study of adult patients admitted to 3 ICUs in the UK and 5 ICUs in the USA. We analysed the haemodynamic changes associated with NOAF. We analysed rate control, rhythm control, and hospital mortality associated with common NOAF treatments. We balanced admission and post-NOAF, pre-treatment covariates across treatment groups.

**Results:**

NOAF was followed by a systolic blood pressure reduction of 5 mmHg (*p* < 0.001). After adjustment, digoxin therapy was associated with inferior rate control versus amiodarone (adjusted hazard ratio (aHR) 0.56, [95% CI 0.34–0.92]). Calcium channel blocker (CCB) therapy was associated with inferior rhythm control versus amiodarone (aHR 0.59 (0.37–0.92). No difference was detected between BBs and amiodarone in rate control (aHR 1.15 [0.91–1.46]), rhythm control (aHR 0.85, [0.69–1.05]), or hospital mortality (aHR 1.03 [0.53–2.03]).

**Conclusions:**

NOAF in ICU patients is followed by decreases in blood pressure. BBs and amiodarone are associated with similar cardiovascular control and appear superior to digoxin and CCBs. Accounting for key confounders removes previously reported mortality benefits associated with BB treatment.

## Highlights

• NOAF in ICU patients is followed by decreases in blood pressure and increased vasoactive drug use
• Beta blockers (BBs) and amiodarone were associated with similar rate and rhythm control
• Digoxin was associated with inferior rate control compared to amiodarone
• Calcium channel blockers were associated with inferior rhythm control compared to amiodarone
• Previously reported mortality benefits associated with BB treatment were not apparent

## 1 Introduction

New-onset atrial fibrillation (NOAF) is a common complication of critical illness, occurring in around 15% of intensive care unit (ICU) admissions [1]. Incidence is higher in certain patient groups, such as those with sepsis [2]. NOAF during critical illness is associated with increased ICU and hospital mortality, and increased length of ICU and hospital stay [1,3]. NOAF during critical illness appears to carry a long-term burden, increasing the risk of future readmissions with heart failure or stroke, and worsening 5-year survival [4]. The haemodynamic impact of NOAF in critically ill patients is poorly understood, but limited data suggests NOAF may precede haemodynamic instability [5].

Guidelines exist for management of AF in patients in the community. However, this is not the case in the critical care setting, where the risks and benefits associated with different treatment options remain unclear, with no large scale trials undertaken [6]. Prior non-randomised studies have adjusted for admission characteristics when comparing treatment groups, but not for physiological status after NOAF. As the haemodynamic changes caused by NOAF may affect initial treatment choice, failure to adjust for these may confound the association between treatment and outcomes [7].

Understandably, there is significant variation within and between units in the management of this common problem [8], demonstrating the need for research to understand the effects of current practice, from which future trials can be designed.

We aimed to describe the characteristics of NOAF and compare the effectiveness of NOAF treatments in patients on an ICU accounting for physiological status after NOAF.

## 2 Methods

### 2.1 Study design

We performed a retrospective observational study of two large intensive care databases from the United Kingdom (UK, Post-Intensive Care Risk-adjusted and Monitoring database (PICRAM) [9,10]) and the United States of America (USA, Medical Information Mart for Intensive Care (MIMIC)-III v1.4 [11]). We published the protocol in advance [12] and report according to the Reporting of studies Conducted using Observational Routinely-collected Data (RECORD) guidelines [13].

### 2.2 Setting

Three general ICUs in the UK from 2008 to 2015 and all ICUs at a tertiary care hospital in the USA between 2001 and 2012.

### 2.3 Data sources

PICRAM comprises data relating to over 12,000 adult patients treated on three general ICUs in the UK from 2008 to 2015. MIMIC-III comprises data on over 40,000 adult patients admitted to five critical care units at a tertiary care hospital in the USA between 2001 and 2012. Study consent and approval details are outlined in Declarations.

### 2.4 Participants

We included all adult (≥16 years) patients treated for NOAF during an ICU stay. For patients admitted more than once to an ICU, we used their first admission.

We excluded patients:

• cared for by a coronary care or cardiac surgery team,
• with missing hospital outcome data,
• with an ICU length of stay (LOS) of less than 24 h [14],
• with significant arrhythmia in the first three hours of arrival to the ICU, or
• with pre-existing arrhythmias

We identified patients with pre-existing arrhythmias where arrhythmia or medications prescribed with an indication of arrhythmia management were present in their medical history.

### 2.5 Variables

Heart rhythm recorded was recorded by the bedside nurse at regular intervals, from a continuous three‑lead electrocardiogram. A documented heart rhythm was assumed to persist until the next identifiable rhythm was recorded. NOAF was defined as the first documented episode of AF lasting at least 30 s [15].

We undertook a scoping review to generate a list of NOAF treatments for analysis [16]. This list was reviewed by our study oversight committee (Additional File 1). Treatments included intravenous (IV) amiodarone, IV beta blockers, IV calcium channel blockers, IV digoxin, and electrical cardioversion. The scoping review also generated a list of potential confounders. The list was then reviewed and supplemented by members of the independent study oversight group (Supplemental Digital Content 1). The final list comprised 30 covariates, comprising 10 admission variables and 20 variables measured after AF onset, prior to treatment.

Vasoactive medications were converted to the vasoactive-inotropic score (VIS) [17].

We analysed each intervention in an intention-to-treat fashion where treatment groups were determined by first treatment after NOAF onset [18].

### 2.6 Primary outcomes

The primary outcomes were time to rate control and time to rhythm control, censored at 24 h. We defined rate control as a heart rate < 110 beats per minute (bpm) in the subset of patients with a heart rate of ≥110 bpm after AF onset [19], and time to rhythm control as the time to first reversion of sinus rhythm in all patients [20]. Patients treated first line with amiodarone were our reference group, as per our published protocol.

### 2.7 Secondary outcome

Our secondary outcome was mortality at 30 days.

### 2.8 Statistical methods

All analyses were performed separately on each database. A combined analysis was performed for each outcome where interventions were present in both databases. All statistical analyses were performed using R Core v4.0.2 [21].

### 2.9 Haemodynamic changes associated with NOAF

We used smooth additive quantile (25%, 50%, 75%) regression models to visualise changes in heart rate, systolic blood pressure (SBP), proportion of patients on vasopressors and VIS during the 6 h before and after NOAF, prior to treatment [22]. We used mixed-effects linear models to test mean changes in haemodynamic variables associated with NOAF. We included separate intercepts and slopes for before / after NOAF as fixed effects, alongside random intercepts and slopes per patient.

### 2.10 Effect of treatment on primary/secondary outcomes

We used weighted Cox regression models to determine the average treatment effect (ATE) for the primary and secondary outcomes, with amiodarone as the reference group. We performed a weighted analysis to adjust for measured confounding between treatment groups.

We used the WeightIt package [23] to generate weightings based on the 30 pre-defined covariates across all treatment groups within each database [24]. We assessed the balance of covariates across weighted groups by tabulating group means pre- and post-weighting and calculating standardised mean differences.

We handled missing values using multiple imputation [25], generating 20 imputed data sets. We resampled each imputed data set with replacement (bootstrapping) 1000 times [26], recalculated the weights and re-fit the Cox models. Mean coefficients and their standard errors were calculated from the empirical distributions of the bootstrap samples for each imputed dataset. Final estimates and standard errors from the 20 imputed data sets were then combined using Rubin's rules [27].

### 2.11 Sensitivity analysis.

Further to assigning weights across all treatment groups, we also repeated the analysis using weights generated in successive pairwise drug comparisons versus amiodarone. We explored unmeasured confounding using array sensitivity analyses [28].

## 3 Results

### 3.1 Study population

**Fig. 1:**
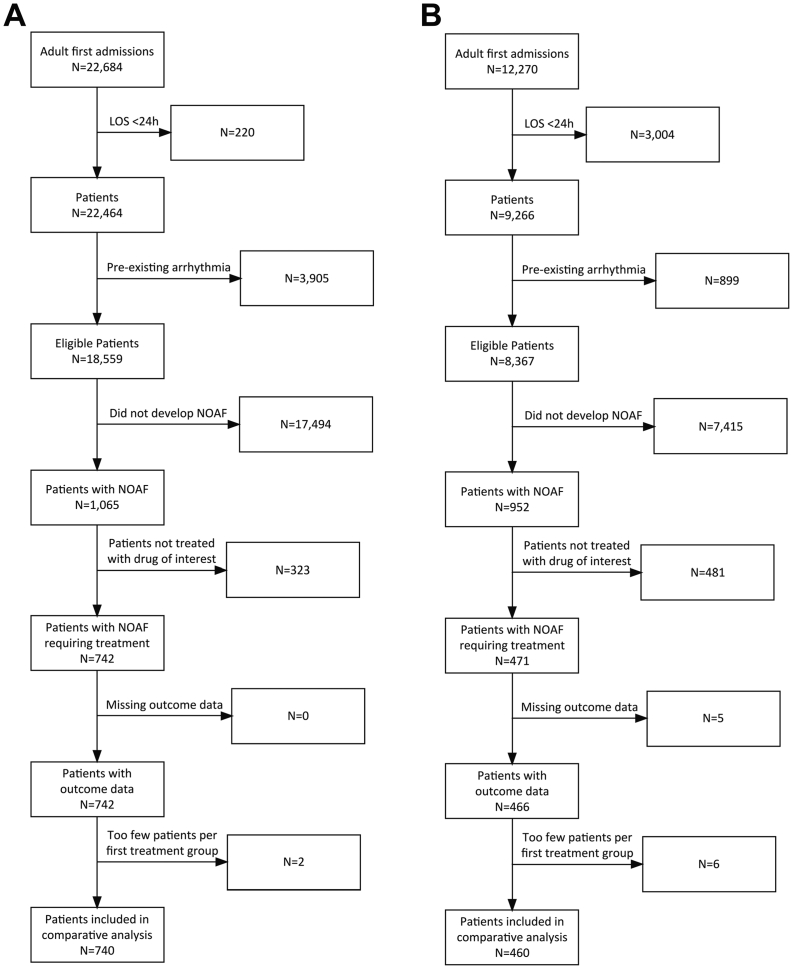
Study consort diagrams for the MIMIC-III database (A) and PICRAM database (B).

The USA database contained 22,684 adult index ICU admissions. Of the 18,559 patients fulfilling our inclusion criteria, 1065 (5.7%) developed NOAF during their ICU admission. Of these, 742 received an intervention of interest. As only two patients receive digoxin as first line treatment, these patients were excluded, leaving 740 patients for the comparative analysis (Fig. 1a).

The UK database contained 12,270 adult index ICU admissions. Of the 8367 patients that fulfilled our inclusion criteria, 952 (11.4%) developed NOAF during their ICU admission. Of these, 466 received an intervention of interest. As only six patients received calcium channel blockers (CCBs) or electrical cardioversion as first line treatment, these patients were excluded, leaving 460 patients for the comparative analysis (Fig. 1b).

The characteristics of included patients are displayed in Table 1. In both databases, patients who developed NOAF tended to be older, had longer ICU and hospital length of stay, and higher ICU and hospital mortality (Supplementary Tables S1 and S2, Additional File 2) compared with patients who had no documentation of AF during ICU admission.

**Table 1:** Characteristics of included patients.

Characteristic	Overall, *N* = 1200	MIMIC-III, *N* = 740	PICRAM, *N* = 460
Age	72 (64, 80)	74 (64, 82)	70 (63, 77)
Sex			
F	558 (46%)	372 (50%)	186 (40%)
M	642 (54%)	368 (50%)	274 (60%)
COPD	116 (9.7%)	53 (7.2%)	63 (14%)
Dialysis-dependent renal failure	8 (0.7%)	1 (0.1%)	7 (1.5%)
NYHA class III/IV heart failure	2 (0.2%)	0 (0%)	2 (0.4%)
Chronic liver disease	31 (2.6%)	11 (1.5%)	20 (4.3%)
Thyroid disorder	61 (5.1%)	33 (4.5%)	28 (6.1%)
Beta blocker therapy prior to admission	344 (30%)	281 (42%)	63 (14%)
Antipsychotic therapy prior to admission	34 (3.0%)	27 (4.0%)	7 (1.5%)
Highest OASIS Score at 3 h	35 (29, 40)	36 (31, 41)	34 (26, 39)
Mechanical ventilation at time of NOAF	586 (49%)	343 (46%)	243 (53%)
Renal replacement therapy during or < 12 h prior to NOAF	112 (9.3%)	47 (6.4%)	65 (14%)
IV Vasoactive medication at time of NOAF	225 (19%)	101 (14%)	124 (27%)
Therapeutic anticoagulation at time of NOAF	84 (7.0%)	36 (4.9%)	48 (10%)
Central venous catheter at time of NOAF	755 (63%)	429 (58%)	326 (71%)
Bronchodilator therapy on day of, or day preceding, NOAF	333 (28%)	258 (35%)	75 (16%)
Plasma sodium concentration (mmol/L)	139.0 (136.0, 142.0)	139.0 (136.0, 143.0)	137.0 (134.0, 141.0)
Plasma potassium concentration (mmol/L)	4.00 (3.80, 4.40)	4.00 (3.70, 4.40)	4.20 (3.90, 4.50)
Plasma magnesium concentration (mmol/L)	0.86 (0.78, 1.00)	0.82 (0.78, 0.95)	0.96 (0.84, 1.12)
Plasma urea concentration (mmol/L)	11 (7, 18)	9 (6, 16)	14 (9, 20)
Plasma creatinine concentration (micromol/L)	104 (69, 186)	97 (62, 159)	125 (78, 214)
White cell count (x10^9^ / L)	12 (8, 16)	12 (8, 16)	11 (8, 16)
Haemoglobin concentration (g/L)	101 (90, 114)	102 (92, 115)	98 (88, 113)
Platelet count (x10^9^ / L)	181 (117, 265)	190 (123, 283)	166 (109, 234)
Prothrombin time (s)	15.0 (13.6, 18.0)	14.2 (13.1, 16.4)	16.1 (15.0, 18.9)
Systolic blood pressure prior to AF onset (mmHg)	120 (104, 138)	124 (106, 142)	114 (101,132)
Mean blood pressure prior to AF onset (mmHg)	77 (68, 89)	78 (68, 90)	75 (68, 88)
Heart rate prior to AF onset (bpm)	95 (83, 109)	92 (81, 101)	102 (88, 118)
Treatment group (by first treatment)			
A miodarone	438 (36.5%)	94 (12.7%)	344 (74.8%)
Beta blocker	520 (43.3%)	473 (63.9%)	47 (10.2%)
Calcium channel blocker	144 (12.0%)	144 (19.5%)	0 (0%)
Digoxin	69 (5.8%)	0 (0%)	69 (15.0%)
Electrical Cardioversion	29 (2.4%)	29 (3.9%)	0 (0%)
ICU Mortality	277 (23.1%)	177 (23.9%)	100 (21.7%)
Hospital mortality	406 (33.8%)	228 (30.8%)	178 (38.7%)

Statistics presented: Median (IQR); n (%).

### 3.2 Characteristics of new-onset atrial fibrillation in treated patients

Time from ICU admission to NOAF onset was similar between the USA and UK databases (median 40 h [IQR 21–79 h] vs 40 h [IQR 41–75 h] respectively) (Supplementary Fig. S1, Additional File 2). Patients in the USA database tended to have shorter total durations of AF (median 12 h [IQR 4–37 h] vs 18 h [IQR 6 - 44 h], respectively) (Supplementary Fig. S2, Additional File 2).

Of the 740 patients in the USA database, 513 (69%) had a heart rate ≥ 110 after AF onset and were eligible for the rate control analysis. Of the 460 patients in the UK database, 318 (69%) had a heart rate ≥ 110 after AF onset and were eligible for the rate control analysis (Supplementary Fig. S3 and S4, Additional File 2).

### 3.3 Haemodynamic changes associated with AF onset

**Fig. 2:**
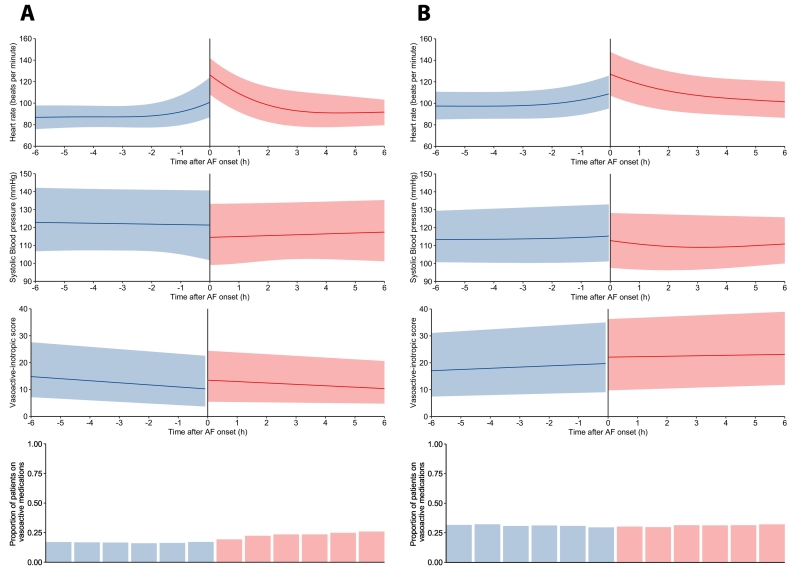
Haemodynamic changes associated with AF onset in the MIMIC-III database (A) and PICRAM database (B). Vasoactive-inotropic score (VIS) shown for those patients receiving vasoactive medications prior to AF onset. VIS = Dopamine dose (mcg/kg/min) + Dobutamine dose (mcg/kg/min) + 100 x Epinephrine dose (mcg/kg/min) + 10 x Milrinone dose (mcg/kg/min) + 10,000 x Vasopressin dose (units/kg/min) + 100 x Norepinephrine dose (mcg/kg/min).

NOAF was associated with a heart rate increase of 22 bpm (*p* < 0.001) and 19 bpm (p < 0.001) in the USA and UK databases, respectively (Fig. 2). NOAF was associated with a significant reduction in systolic blood pressure (SBP) of 7 mmHg and 4 mmHg in the USA and UK databases, respectively (5 mmHg in combined analysis) (p < 0.001). This was despite increases in the doses of vasoactive medication after NOAF onset in those receiving vasoactive medications prior to NOAF onset (VIS increase of 2.5 (p < 0.001) and 1.9 (*p* = 0.001) in the USA and UK databases, respectively (2.3 in combined analysis). New hypotension (SBP <90 or MBP <65) occurred after NOAF in 28% and 21% of patients with SBP ≥90 and MBP ≥65 prior to AF onset in the USA and UK databases. There was no significant change in the proportion of patients receiving vasoactive medications after NOAF onset 17.6% to 20.2%, *p* = 0.29 or 30.1% to 31.1% *p* = 0.98 in the USA and UK databases, respectively.

### 3.4 NOAF treatment groups

In patients identified in the USA database, 94 received amiodarone, 473 received beta blockers (metoprolol, esmolol or labetalol), 144 received CCBs (diltiazem or verapamil), and 29 received electrical cardioversion as their initial NOAF treatment. In the UK database, 344 patients received amiodarone, 47 received beta blockers, and 69 received digoxin as their initial NOAF treatment (Supplementary Tables S3 and S4, Additional File 2).

Physiological status immediately after NOAF onset differed across treatment groups. Patients treated with beta blockers tended to have higher blood pressure and lower vasoactive drug use than patients treated with amiodarone (USA database: median systolic blood pressure (SBP) 119 mmHg vs 103mmhg, median mean blood pressure (MBP) 80 mmHg vs 72 mmHg, proportion of patients on vasoactive medications 9.5% vs 36%; UK database: median SBP 128 mmHg vs 112 mmHg, median MBP 83 mmHg vs 74 mmHg, proportion of patients on vasoactive medications 13% vs 31%). Patients who received electrical cardioversion (all in the USA database) were the least cardiovascularly stable (median SBP 93 mmHg, median MBP 67 mmHg, proportion of patients on vasoactive medications 45%) (Supplementary Tables S3 and S4, Additional File 2).

### 3.5 Adjustment for confounding

After propensity score weighting, covariates were well matched across all treatment groups in each database and in the combined dataset (Supplementary Tables S5, S6 and S7, Additional File 2). The distribution of weights was comparable across treatment groups (Supplementary Fig. S15, S16 and S17, Additional File 2). Missing data proportions per variable are shown in Supplementary tables S11 and S12, Additional File 2.

### 3.6 Outcomes

Unadjusted time-to-event curves are shown in Supplementary Fig. S5-14, Additional File 2. Unadjusted and adjusted hazard ratios for each outcome in the USA, UK, and combined databases are shown in Supplementary Tables S8, S9 and S10, respectively, Additional File 2.

#### 3.6.1 Rate control

**Fig. 3:**
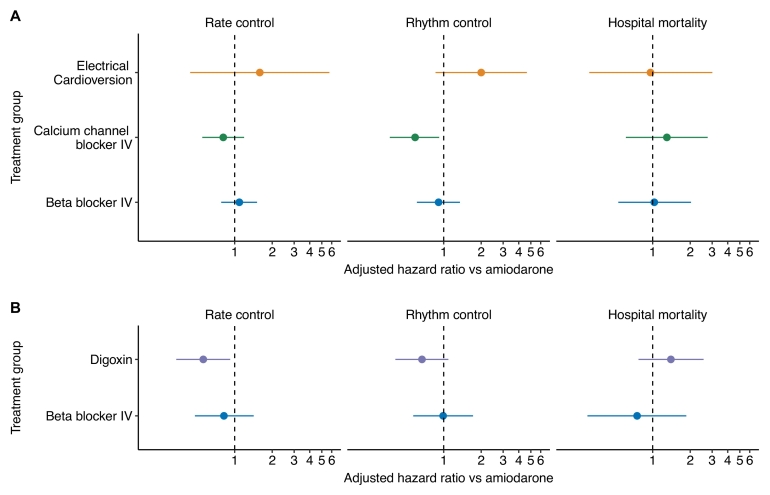
Adjusted hazard ratios (compared with amiodarone) for rate and rhythm control by treatment group.

Digoxin therapy was associated with inferior rate control (adjusted hazard ratio (aHR) 0.56 [95% CI 0.34–0.92]) all in the UK database (Fig. 3) in comparison to amiodarone.

No differences in achieving rate control were observed with beta blockers (aHR 1.09 [0.78–1.51]), CCBs (aHR 0.81 [0.55–1.19]) or cardioversion (aHR 1.59 [0.44–5.75]) versus amiodarone in the USA database, or beta blockers versus amiodarone in the UK database (aHR 0.82 [0.48–1.42]).

In the combined database analysis of beta blockers versus amiodarone, rate control with beta blockers appeared superior in the unadjusted analysis, however, after adjustment, these differences were no longer evident (aHR 1.15 [0.91–1.46]), consistent with our individual database analyses (Supplementary Table S10, Additional File 2).

#### 3.6.2 Rhythm control

CCBs were associated with inferior rhythm control (aHR 0.59 [0.37–0.92]) versus amiodarone in the USA database (Fig. 3). No difference was identified in rhythm control with beta blockers (aHR 0.91 [0.61–1.35]) or cardioversion (2.00 [CI 0.86–4.65]) versus amiodarone in the USA database.

In the UK database, digoxin was not significantly associated with inferior rhythm control (aHR 0.67 [0.41–1.09]) versus amiodarone. As in the USA database, beta blocker therapy was not associated with any significant difference in time to rhythm control (aHR 0.99 ([0.57–1.72]).

In the combined database analysis of beta blockers versus amiodarone, rhythm control with amiodarone appeared superior in the unadjusted analysis, however, after adjustment, the difference was no longer evident (aHR 0.85, [0.69–1.05]), consistent with our individual database analyses (Supplementary Table S10, Additional File 2).

#### 3.6.3 Hospital mortality

We found no significant differences between beta blockers (aHR 1.03 [0.53–2.03]), CCBs (aHR 1.30 [0.61–2.76)), or electrical cardioversion (aHR 0.96 [0.31–3.01]) in hospital mortality versus amiodarone in the USA database. Similarly, no differences were identified between beta blockers (aHR 0.75 [0.30–1.86]) or digoxin (aHR 1.40 [0.77–2.56]) versus amiodarone in hospital mortality in the UK database.

Beta blockers therapy appeared to be associated with reduced mortality in the combined dataset prior to adjustment, however, after adjustment, the difference was no longer evident (aHR 1.03 [0.53–2.03]), consistent with our individual database analyses ((Supplementary Table S10, Additional File 2).

### 3.7 Sensitivity analysis

Our findings remained in the sensitivity analysis (CCB rhythm control aHR 0.55 [0.37–0.82], digoxin rate control aHR 0.56 [0.36–0.88]). Furthermore, CCB therapy was associated with an increased risk of reversion to a heart rate > 110 (aHR 1.82 [1.10–3.00]) (Additional File 3).

Array sensitivity analysis suggested that an unmeasured confounder would require high imbalance between treatment groups and extreme association with outcome to bring the observed effect estimate to the null for digoxin's inferior rate control and CCB's inferior rhythm control (Supplementary Tables S11 and S12, Additional File 3).

## 4 Discussion

We investigated the characteristics of NOAF in patients on an ICU and outcomes associated with first-line treatments. We balanced admission covariates plus pre-treatment physiological variables after NOAF onset across multiple treatment groups in American and UK databases. NOAF was associated with an increase in heart rate, and a decrease in blood pressure despite an increase in vasoactive medication doses. Digoxin therapy was associated with inferior rate control compared with amiodarone. Calcium channel blocker therapy was associated with inferior rhythm control compared with amiodarone (and increased reversion to AF in sensitivity analysis). Preference for first-line treatment differed between the databases, suggesting different treatment paradigms between the UK and the USA.

Although previous evidence for the cardiovascular effects of NOAF in ICU patients is limited, our findings are not surprising. Organised atrial activity contributes to ventricular filling and cardiac output [29]. NOAF precludes these mechanisms and the effects of the loss of atrial contraction on ventricular filling may be compounded by the diastolic dysfunction commonly seen during critical illness [30].

Digoxin is known to be less effective during states of increased sympathetic drive [31] and catecholaminergic medication use [32]. Its slower onset of action is also recognised [33]. These characteristics may explain our findings of inferior performance in comparison to amiodarone in patients treated on an ICU.

We found that CCBs were associated with inferior rhythm control versus amiodarone. Our findings contrast with one RCT identified which compared amiodarone with CCBs [34] and found no difference in achieving rhythm control. However, this study included only 20 patients per treatment group so was potentially underpowered. A small (*n* = 24) randomised study of paroxysmal AF outside ICU reported a cardioversion proportion of 0% in patients who received verapamil vs 77% in patients who received amiodarone [35]. While electrical cardioversion resulted in a faster return to sinus rhythm than other therapies, this result was not statistically significant, possibly due to the small number of patients in this treatment group.

In the USA and combined database our unadjusted analysis suggested a mortality benefit in the beta blocker treatment group. However, after adjustment, there was no mortality difference. Our adjusted results conflict with one large study which suggested a survival benefit with beta blockers over amiodarone therapy in patients with sepsis [7]. However, this study was unable to adjust for physiological status at the time of NOAF onset before treatment. Our study shows that patients in whom clinicians chose beta blocker treatment tended to be more cardiovascularly stable than those in whom amiodarone was selected. Failure to adjust for these factors may result in residual confounding. Rate and rhythm control were similar with beta-blockers and amiodarone. These findings are consistent with a previous study of patients in ICU with new or pre-existing AF that found no difference in time to rate control between metoprolol and amiodarone [36].

Our analysis has several strengths. First, we performed comprehensive adjustment of variables likely to influence treatment choice, including physiological variables after AF onset. We show significant differences in post-AF pre-treatment variables according to the therapy used. Furthermore, the scale of our study provided a sample size large enough to demonstrate differential efficacy in NOAF treatments.

Our analysis also has limitations. NOAF was not independently verified. Documentation of AF in the MIMIC-III database has, however, been shown to be accurate for determining AF onset to within 1 h of true onset as determined by independent review of ECG waveforms [37]. Documentation of comorbidities in the MIMIC-III database relied on hospital billing codes which may not have identified all cases. While some patients with pre-existing AF therefore may not have been identified, we mitigated this by excluding patients with AF recorded during their initial hours in the ICU. Furthermore, the study was retrospective and not randomised. We are unable to exclude bias introduced by residual unmeasured confounding. However, variables likely to influence treatment choice were felt to be well represented. We also explored unmeasured confounding using array sensitivity analyses which revealed that an unmeasured variable would need to be highly imbalanced between the CCB or digoxin group and the amiodarone group and have a very strong association with time to rhythm or rate control, respectively, to bring the effect estimate to the null. Our data was not complete for every variable, but missing data proportions were low. We used multiple imputation to limit bias introduced by this missing data. Additionally, the time frames differed between the MIMIC-III and PICRAM databases which may limit the interpretation of the combined analysis. Lastly, our findings should be interpreted in the context of undifferentiated ICU patients; certain treatments including CCBs and digoxin may be highly appropriate in certain clinical scenarios.

Despite these limitations, our work provides some of the most detailed information on the effects of current treatments for NOAF in patients on an ICU to date.

## 5 Conclusions

Beta blockers or amiodarone would appear better first line therapies than digoxin or calcium antagonists for the management of new-onset atrial fibrillation in undifferentiated patients treated on an ICU. Previously reported mortality benefits with beta-blockers, which we also found on unadjusted analyses, are not present when admission variables, laboratory variables, and physiological variables after AF onset were accounted for. Our work may guide future randomised trials of NOAF treatments. However, these would require careful planning to identify where certain treatments may be of more benefit in clinical subgroups of a heterogenous population.

## Author statement

JB extracted data for the scoping review, extracted data from the PICRAM database, performed statistical analysis on data from the MIMIC-III an PICRAM databases, and co-wrote the manuscript. JB is the guarantor of the paper. AJ extracted data from the MIMIC-III database, and synthesised data from both databases. OR provided contributed to data analysis. SG provided statistical support and contributed to data analysis. JD contributed to conception and supervision of the analysis and to manuscript preparation. Kim Rajappan provided expert review and advice on atrial fibrillation/arrhythmias. PM (Project Management at ICNARC) contributed to study conception, study management and database analyses. DH contributed to study conception and contributed to the acquisition, analysis, and interpretation of data. DY was involved in study design, data extraction specifications, and interpretation of the results. He was the Chief Investigator for the PICRAM study, which provided one of the databases for the CAFÉ study. KR contributed to study conception and design. PW contributed to study conception, design and data analysis and co-wrote the manuscript.

## Funding

This study was funded by a 10.13039/501100000272National Institute for Health Research, United Kingdom (NIHR), Health Technology Assessment programme grant 17/71/04.

JB is supported by an NIHR Doctoral Research Fellowship (NIHR300224). OR is supported by a Drayson Research Fellowship. PW is supported by the Oxford 10.13039/100014461Biomedical Research Centre.

## Notation of prior abstract publication/presentation

None.

## Ethics approval and consent to participate

The PICRAM study was granted ethical approval by the NRES Committee Oxford (ref: 11/SC/0440) and the National Information Governance Board (ref: ECC 7–05(f)/2011). Use of patient data without informed consent was approved by the National Information Governance Board on 2nd February 2012 (ref: ECC 7-05(f)/2011). The MIMIC-III database and associated use of patient data was approved by the Institutional Review Boards of Beth Israel Deaconess Medical Center (Boston, MA) and the Massachusetts Institute of Technology (Cambridge, MA).

## Consent for publication

N/A

## Availability of data and materials

MIMIC-III is an openly available dataset (details at https://mimic.physionet.org/). The PICRAM dataset is not currently publicly available.

## Authors' contributions

JB extracted data for the scoping review, extracted data from the PICRAM database, performed statistical analysis on data from the MIMIC-III an PICRAM databases, and co-wrote the manuscript. JB is the guarantor of the paper. AJ extracted data from the MIMIC-III database, and synthesised data from both databases. OR provided contributed to data analysis. SG provided statistical support and contributed to data analysis. JD contributed to conception and supervision of the analysis and to manuscript preparation. KR provided expert review and advice on atrial fibrillation/arrhythmias. PM contributed to study conception, study management and database analyses. DH contributed to study conception and contributed to the acquisition, analysis and interpretation of data. DY was involved in study design, data extraction specifications, and interpretation of the results. He was the Chief Investigator for the PICRAM study, which provided one of the databases for the CAFÉ study. KR contributed to study conception and design. PW contributed to study conception, design and data analysis and co-wrote the manuscript.

## Declaration of Competing Interest

PW worked part time for Sensyne Health and has received grant funding from National Institute for Health Research, and Sensyne Health outside the submitted work. PW was a member of the HTA EESC Methods Group 2014–2017, HTA EESC Panel 2013–2018, HTA Prioritisation Committee B (In hospital) 2018–2019 and NIHR i4i PDA A Committee 2018-. KR was a member of the DHSC/UKRI COVID-19 Rapid Response Research Initiative Member, College of Experts 2020, HS&DR Funding Committee Member 2014–2019, HS&DR Rapid Service Evaluation Themed Call Committee Member 2017, HS&DR Commissioned - Board Member 2016, and HTA Trauma Themed Call Board Member 2007–2008. DY received a salary from the University of Oxford during the duration of the work.

## Appendix A Supplementary data

Supplementary material 1 Image 1

Supplementary material 2 Image 2

Supplementary material 3 Image 3
