# Supplementary material for: Comparative effectiveness of common treatments for new-onset atrial fibrillation within the ICU: Accounting for physiological status
Source: J Crit Care. 2022 Feb;67:149–56. doi: 10.1016/j.jcrc.2021.11.005 (PMC8687206; doi:10.1016/j.jcrc.2021.11.005)
Supplement: Supplementary file 1 — Supplementary material 1 [file mmc1.pdf]

Additional File 1
Expert panel details and research recommendations

Name	Role	Institution
**Professor Peter Watkinson**	Associate professor of intensive care medicine	University of Oxford
**Dr Jonathan Bedford**	Clinical Research Fellow	University of Oxford
**Dr Andrew Walden**	Consultant in intensive care medicine	Intensive care unit, Royal Berkshire Hospital
**Professor Ben O’Brien**	Professor of Perioperative Medicine	St Bartholomew's Hospital & Barts Heart Centre
**Kim Rajappan**	Consultant in cardiology	Oxford University Hospitals NHS Foundation Trust
**Ian Taylor**	Lay representative	N/A
**Cathy Taylor**	Lay representative	N/A

Treatments and confounding variables

The following lists were drawn initially from the scoping review, then refined and ratified by the expert panel.

1 Treatments of interest

1.1 Treatments to be included in the analysis

- **Amiodarone IV**
- **Beta blockers IV**
    - Labetalol
    - Esmolol
    - Metoprolol
- **Calcium channel blockers IV**
    - Diltiazem
    - Verapamil
- **Digoxin IV**
- **Electrical cardioversion**

Treatments of interest but not possible with our data

Propafenone and ibutilide: identified as candidate therapies in scoping review, however these were not available in our datasets.

2 Confounding / matching variables

2.1 Demographic / Comorbid factors

- Age
- Sex
- Congestive cardiac failure
- COPD (previous or current diagnosis)
- Liver disease (previous or current diagnosis)
- Chronic renal failure
- Thyroid disorders (previous or current diagnosis, or taking relevant medications)
- Pre-admission beta blockers
- Pre-admission antipsychotic medication

2.2 Admission factors

- Illness severity in first 3h (therefore not influenced by NOAF (as we are excluding pts in AF in first 3h)).

2.3 Factors at the time of NOAF onset

- Heart rate[o]
- Blood pressure[o]
- Body temperature[o]
- Presence of vasopressor / inotrope[o]
- Dose of noradrenaline[o]
- Dose of vasopressin[o]
- White cell count[L]
- Plasma electrolyte (K, Mg, Na, Ca) concentrations[L]
- Plasma urea and creatinine concentrations[L]
- Platelet count[L]
- Prothrombin time[L]
- Presence of therapeutic dose anticoagulation*
- Presence of bronchodilator therapy*
- Mechanical ventilation[%]
- Haemofiltration[$]
- Presence of central venous access[%]

[o]First recording after AF onset

[L]Most recent value in ICU prior to AF onset

*On the calendar day of, or the day preceding NOAF onset

[%]At the time of NOAF onset
