# Supplementary material for: Comparative effectiveness of common treatments for new-onset atrial fibrillation within the ICU: Accounting for physiological status
Source: J Crit Care. 2022 Feb;67:149–56. doi: 10.1016/j.jcrc.2021.11.005 (PMC8687206; doi:10.1016/j.jcrc.2021.11.005)
Supplement: Supplementary file 2 — Supplementary material 2 [file mmc2.pdf]

Additional File 2

Supplementary data

Contents

Study population ... 4

Supplemental Table S1 - Characteristics of patients with NOAF vs without NOAF: MIMIC database ... 4

Supplemental Table S2 - Characteristics of patients with NOAF vs those without: PICRAM database ... 4

Supplemental Figure S1 - Time from ICU admission to NOAF onset in treated patients. ... 5

Supplemental Figure S2 - Total duration of AF per treated patient. ... 6

Rate control ... 6

Supplemental Figure S3 - Distribution of heart rate post AF onset: MIMIC-III database ... 7

Supplemental Figure S4 - Distribution of heart rate post AF onset: MIMIC-III database ... 8

NOAF treatments ... 9

Supplemental Table S3 - Characteristics of included patients by treatment group: MIMIC-III database ... 9

Supplemental Table S4 - Characteristics of included patients by treatment group: PICRAM database ... 10

Supplemental Figure S5 - Cumulative incidence plot of time from treatment to heart rate <110 for each treatment: MIMIC database ... 12

Supplemental Figure S6 - Cumulative incidence plot of time from treatment to heart rate <110 for each treatment: PICRAM database ... 12

Supplemental Figure S7 - Kaplan-Meier curves of time from achieving rate control to reversion to heart rate ≥110: MIMIC-III database ... 13

Supplemental Figure S8 - Kaplan-Meier curves of time from achieving rate control to reversion to heart rate ≥110: PICRAM database ... 13

Supplemental Figure S9 - Cumulative incidence plot of time from treatment to rhythm control for each treatment: MIMIC database ... 14

Supplemental Figure S10 - Cumulative incidence plot of time from treatment to rhythm control for each treatment: PICRAM database 14

Supplemental Figure S11 - Kaplan-Meier curves for each treatment of time from achieving rhythm control to reversion to AF: MIMIC-III database 15

Supplemental Figure S12 - Kaplan-Meier curves for each treatment of time from achieving rhythm control to reversion to AF: PICRAM database 15

Survival 16

Supplemental Figure S13 - Kaplan-Meier survival curves for each treatment group: MIMIC-III database 16

Supplemental Figure S14 - Kaplan-Meier survival curves for each treatment group: PICRAM database 16

Adjustment for confounding 17

Supplemental Table S5 - Unweighted and weighted covariate means by treatment group: MIMIC-III database 18

Supplemental Table S6 - Unweighted and weighted covariate means by treatment group: PICRAM database 20

Supplemental Table S7 - Unweighted and weighted covariate means by treatment group: Combined databases 23

Supplemental Figure S15 - Histograms of propensity weights by treatment group: MIMIC-III database 25

Supplemental Figure S16 - Histograms of propensity weights by treatment group: PICRAM database 25

Supplemental Figure S17 - Histograms of propensity weights by treatment group: Combined dataset 26

Outcomes 27

Supplemental Table S8 - Unadjusted and adjusted hazard ratios for each outcome (compared with amiodarone) and associated 95% confidence intervals: MIMIC-III database 27

Supplemental Table S9 - Unadjusted and adjusted hazard ratios (compared with amiodarone) for each outcome and associated 95% confidence intervals: PICRAM database 27

Supplemental Table S10 - Unadjusted and adjusted hazard ratios for each outcome (compared with amiodarone) and associated 95% confidence intervals: Combined databases 28

Missingness of variables 28
Supplemental Table S11 - Missingness of weighting variables: MIMIC-III database 28
Supplemental Table S12 - Missingness of matching variables: PICRAM database 28

Study population

Supplemental Table S1 - Characteristics of patients with NOAF vs without NOAF: MIMIC database

Characteristic	Without NOAF, N = 17,494	NOAF, N = 1,065
Age	59 (47, 73)	75 (64, 83)
Sex		
F	8,440 (48%)	518 (49%)
M	9,054 (52%)	547 (51%)
Weight (Kg)	77 (64, 91)	77 (65, 92)
ICU length of stay (d)	1.8 (1.0, 3.5)	5.8 (3.0, 11.8)
ICU mortality	1,260 (7.2%)	265 (25%)
Hospital length of stay (d)	6 (4, 12)	12 (7, 21)
Hospital mortality	1,887 (11%)	347 (33%)

Continuous variables are summarised as Median (IQR); Categorical variables are summarised as n (%)

Supplemental Table S2 - Characteristics of patients with NOAF vs those without: PICRAM database

Characteristic	Never AF, N = 7,415	NOAF, N = 952
Age	61 (45, 71)	71 (64, 78)
Sex		
F	3,120 (42%)	368 (39%)
M	4,295 (58%)	584 (61%)
Weight (Kg)	72 (62, 83)	75 (65, 85)
ICU length of stay (d)	2.4 (1.5, 4.7)	6.1 (3.1, 12.8)
ICU mortality	628 (8.5%)	193 (20%)
Hospital length of stay (d)*	13 (7, 25)	19 (10, 40)
Hospital mortality	1,122 (16%)	350 (37%)

Continuous variables are summarised as Median (IQR); Categorical variables are summarised as n (%).

*32 patients for whom hospital length of stay unknown not included.

Characteristics of new-onset atrial fibrillation in treated patients

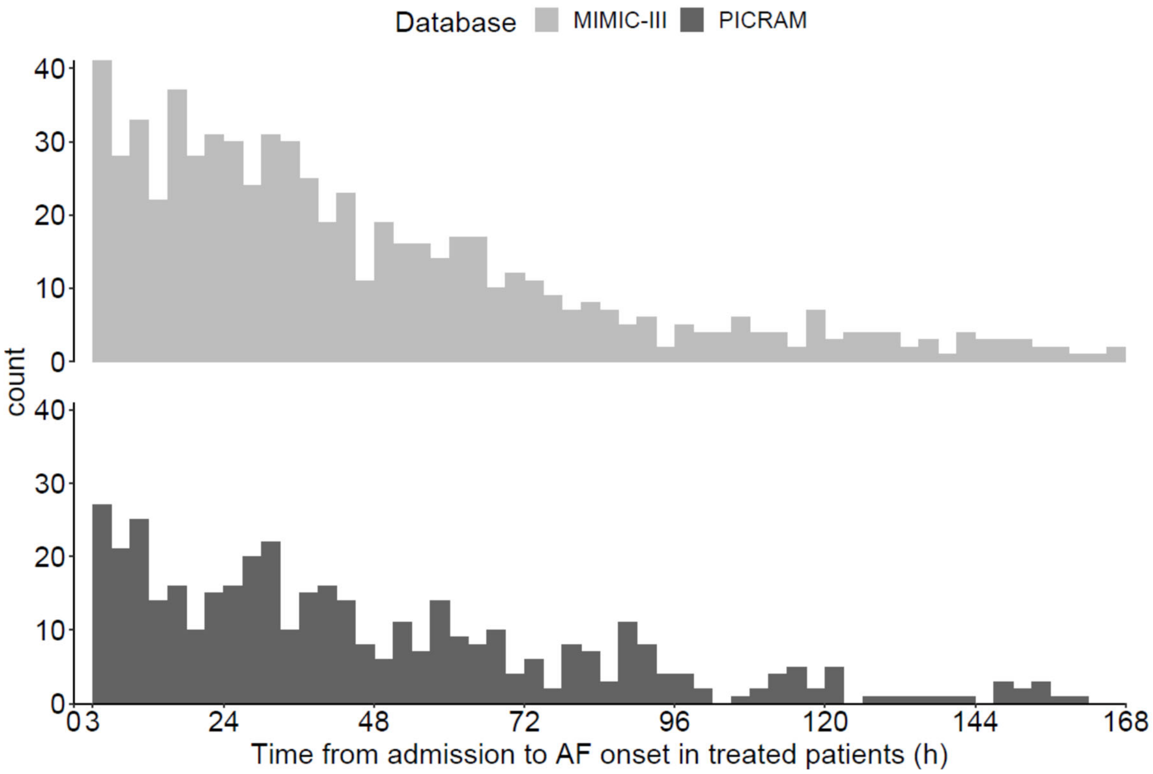

Supplemental Figure S1 - Time from ICU admission to NOAF onset in treated patients.

Data from 93 patients with time to AF onset >168h (7 days) not shown

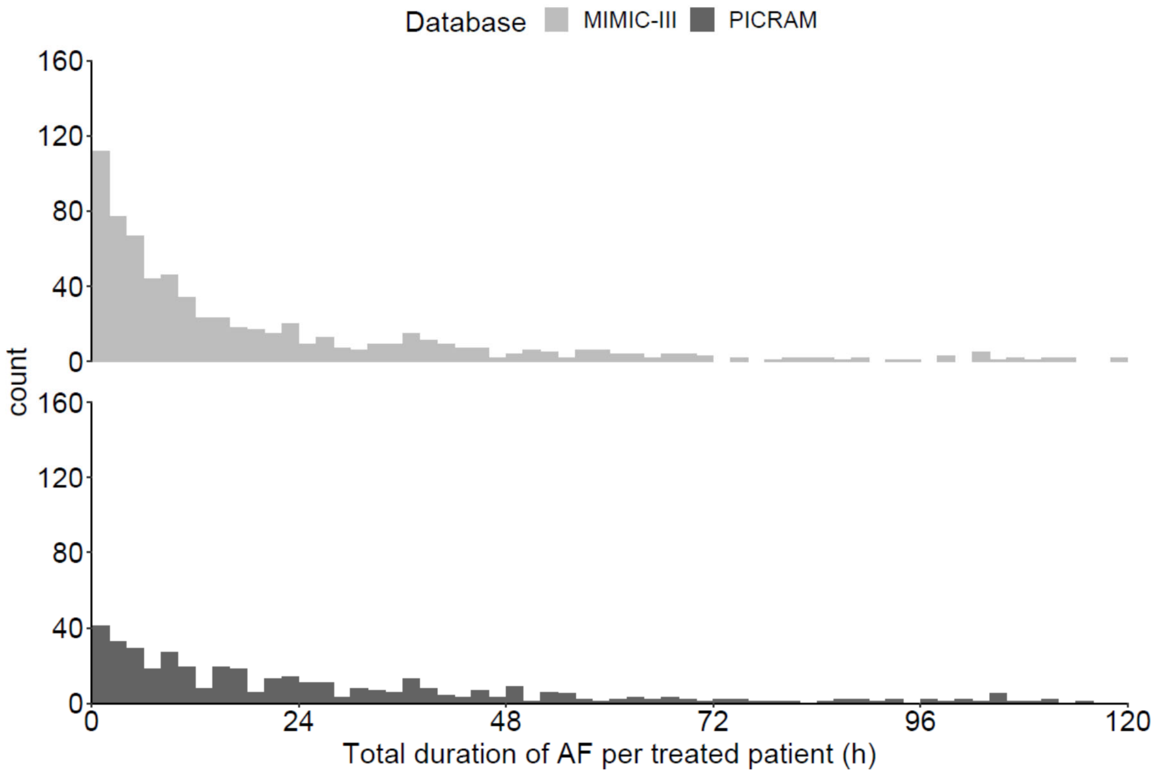

Supplemental Figure S2 - Total duration of AF per treated patient.

Data from 89 patients with AF duration >120h (5 days) not shown

Rate control

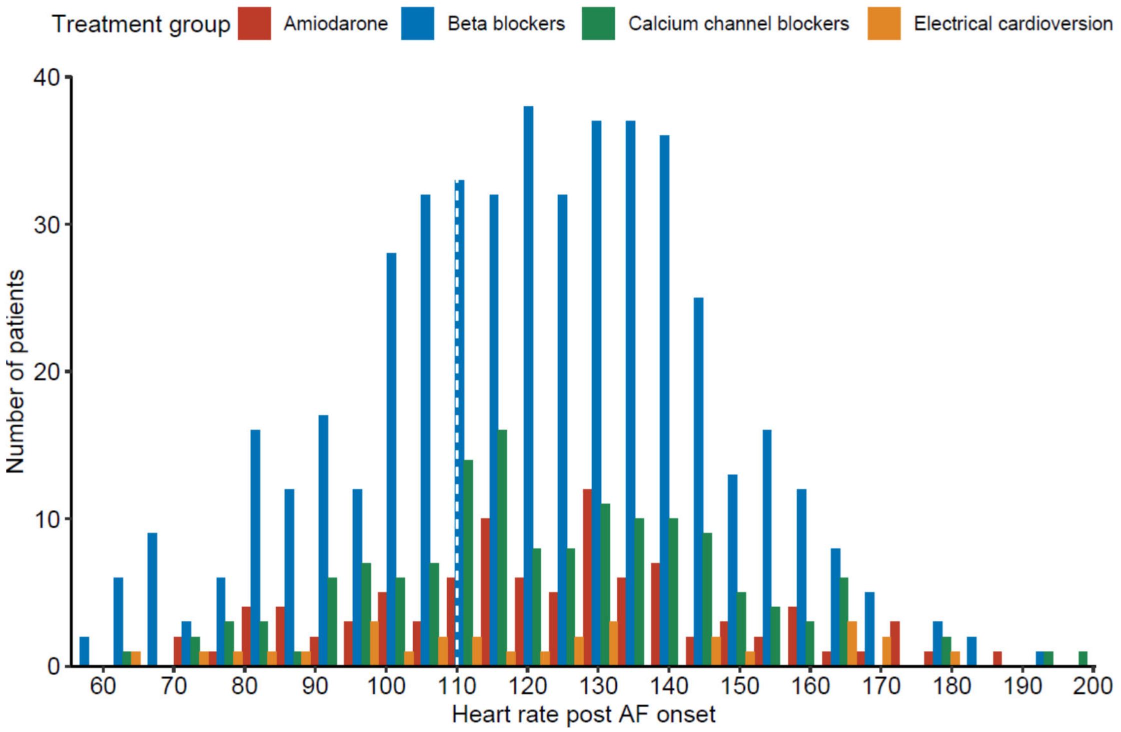

Supplemental Figure S3 - Distribution of heart rate post AF onset: MIMIC-III database

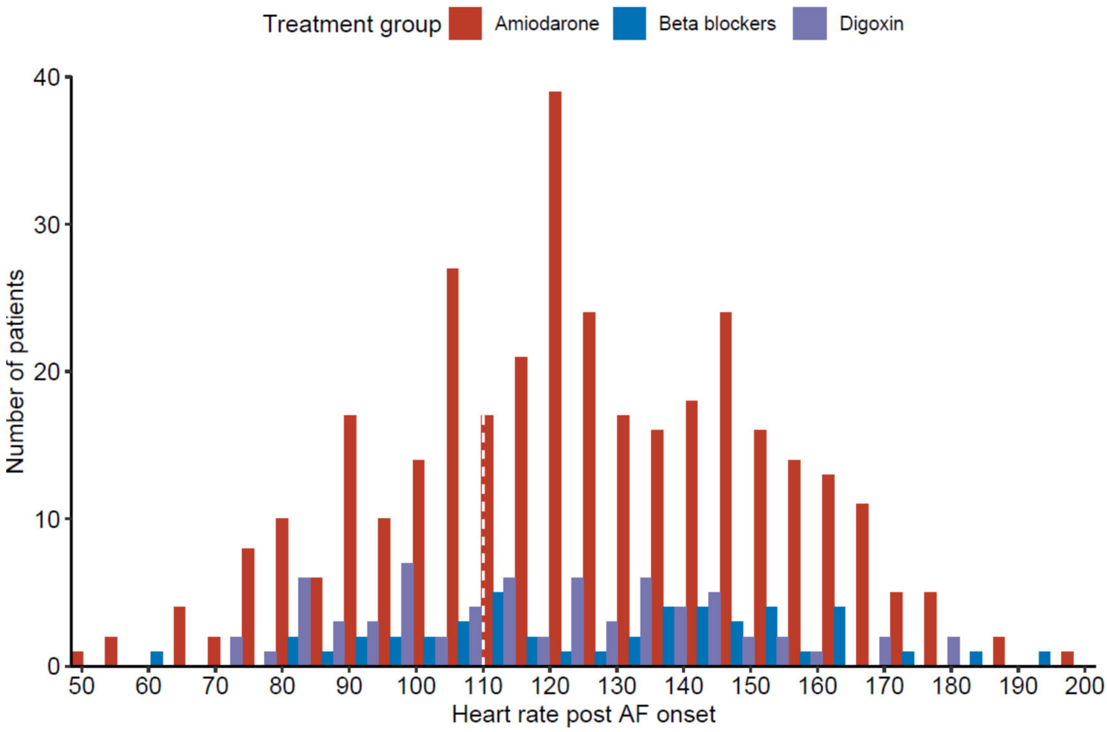

Supplemental Figure S4 - Distribution of heart rate post AF onset: MIMIC-III database

NOAF treatments

Supplemental Table S3 - Characteristics of included patients by treatment group: MIMIC-III database

Characteristic	Overall, N = 740	Amiodarone, N = 94	Beta blocker, N = 473	Calcium channel blocker, N = 144	DC cardioversion, N = 29
Age	74 (64, 82)	76 (63, 83)	73 (64, 83)	73 (65, 81)	77 (69, 85)
Sex					
F	372 (50%)	51 (54%)	234 (49%)	77 (53%)	10 (34%)
M	368 (50%)	43 (46%)	239 (51%)	67 (47%)	19 (66%)
COPD	53 (7.2%)	3 (3.2%)	28 (5.9%)	19 (13%)	3 (10%)
NYHA class III/IV heart failure	0 (0%)	0 (0%)	0 (0%)	0 (0%)	0 (0%)
Dialysis-dependent renal failure	1 (0.1%)	0 (0%)	1 (0.2%)	0 (0%)	0 (0%)
Chronic liver disease	11 (1.5%)	2 (2.1%)	7 (1.5%)	2 (1.4%)	0 (0%)
Thyroid disorder	33 (4.5%)	2 (2.1%)	27 (5.7%)	3 (2.1%)	1 (3.4%)
Beta blocker therapy prior to admission	281 (42%)	38 (46%)	193 (45%)	37 (28%)	13 (52%)
Antipsychotic medication prior to admission	27 (4.0%)	4 (4.8%)	19 (4.4%)	4 (3.1%)	0 (0%)
Highest OASIS Score at 3h	36 (31, 41)	38 (32, 43)	36 (31, 40)	36 (30, 40)	40 (33, 43)
Mechanical ventilation at time of NOAF	343 (46%)	60 (64%)	206 (44%)	55 (38%)	22 (76%)
Renal replacement therapy during or <12h prior to NOAF	47 (6.4%)	5 (5.3%)	38 (8.0%)	1 (0.7%)	3 (10%)
IV Vasoactive medication at time of NOAF	101 (14%)	34 (36%)	45 (9.5%)	9 (6.2%)	13 (45%)
Therapeutic anticoagulation at time of NOAF	36 (4.9%)	6 (6.4%)	23 (4.9%)	5 (3.5%)	2 (6.9%)
Central venous catheter at time of NOAF	429 (58%)	69 (73%)	261 (55%)	75 (52%)	24 (83%)
Bronchodilator therapy on day of, or day preceeding, NOAF	258 (35%)	33 (35%)	155 (33%)	62 (43%)	8 (28%)
Plasma sodium concentration (mmol/L)	139 (136, 143)	138 (136, 141)	140 (137, 143)	140 (137, 143)	138 (136, 143)

Plasma potassium concentration (mmol/L)	4.0 (3.7, 4.4)	4.0 (3.7, 4.5)	3.9 (3.6, 4.3)	4.0 (3.7, 4.3)	4.1 (3.7, 4.4)
Plasma magnesium concentration (mmol/L)	0.82 (0.78, 0.95)	0.86 (0.78, 0.95)	0.82 (0.78, 0.95)	0.86 (0.78, 0.95)	0.82 (0.78, 0.91)
Plasma urea concentration (mmol/L)	9.3 (6.1, 16.1)	10.0 (6.4, 15.4)	8.9 (5.7, 15.7)	8.9 (6.1, 15.4)	19.8 (9.5, 23.8)
Plasma creatinine concentration (micromol/L)	97 (62, 159)	97 (71, 168)	97 (62, 159)	88 (62, 139)	159 (86, 270)
White cell count (x109 / L)	11.8 (8.5, 16.1)	13.6 (8.8, 19.7)	11.6 (8.8, 15.5)	11.3 (7.5, 16.3)	13.1 (10.5, 16.3)
Haemoglobin concentration (g/L)	102 (92, 115)	100 (88, 113)	104 (92, 115)	101 (93, 116)	99 (91, 111)
Platelet count (x109 / L)	190 (123, 283)	179 (91, 258)	190 (129, 286)	205 (137, 291)	161 (111, 219)
Prothrombin time (s)	14.2 (13.1, 16.4)	15.2 (13.7, 17.8	14.2 (13.1, 16.3)	14.0 (12.9, 15.6)	15.0 (13.6, 17.5)
Systolic blood pressure after AF onset	116 (100, 135)	103 (93, 122)	119 (104, 140)	115 (97, 132)	93 (88, 111)
Mean blood pressure after AF onset	78 (67, 90)	72 (63, 81)	80 (69, 92)	76 (66, 88)	67 (61, 78)
Heart rate after AF onset	122 (104, 137)	124 (110, 139)	121 (102, 136)	124 (110, 141)	123 (98, 147)

Supplemental Table S4 - Characteristics of included patients by treatment group: PICRAM database

**Characteristic**	**Overall, N = 460**	**Amiodarone, N = 344**	**Beta blocker, N = 47**	**Digoxin, N = 69**
Age	70 (63, 77)	69 (63, 77)	70 (64, 76)	75 (65, 81)
Sex				
F	186 (40%)	141 (41%)	20 (43%)	25 (36%)
M	274 (60%)	203 (59%)	27 (57%)	44 (64%)
COPD	63 (14%)	51 (15%)	1 (2.1%)	11 (16%)
NYHA class III/IV heart failure	2 (0.4%)	2 (0.6%)	0 (0%)	0 (0%)
Dialysis-dependent renal failure	7 (1.5%)	6 (1.7%)	0 (0%)	1 (1.4%)
Chronic liver disease	20 (4.3%)	14 (4.1%)	1 (2.1%)	5 (7.2%)
Thyroid disorder	28 (6.1%)	21 (6.1%)	3 (6.4%)	4 (5.8%)
Beta blocker therapy prior to admission	63 (14%)	44 (13%)	10 (21%)	9 (13%)
Antipsychotic medication prior to admission	7 (1.5%)	5 (1.5%)	1 (2.1%)	1 (1.4%)
Highest OASIS Score at 3h	34 (26, 39)	34 (27, 40)	34 (22, 38)	30 (25, 36)
Mechanical ventilation at time of NOAF	243 (53%)	192 (56%)	22 (47%)	29 (42%)
Renal replacement therapy during or <12h prior to NOAF	65 (14%)	52 (15%)	5 (11%)	8 (12%)

IV Vasoactive medication at time of NOAF	124 (27%)	105 (31%)	6 (13%)	13 (19%)
Therapeutic anticoagulation at time of NOAF	48 (10%)	37 (11%)	5 (11%)	6 (8.7%)
Central venous catheter at time of NOAF	326 (71%)	262 (76%)	32 (68%)	32 (46%)
Bronchodilator therapy on day of, or day preceeding, NOAF	75 (16%)	57 (17%)	7 (15%)	11 (16%)
Plasma sodium concentration (mmol/L)	137 (134, 141)	137 (134, 141)	139 (136, 144)	138 (135, 140)
Plasma potassium concentration (mmol/L)	4.2 (3.9, 4.5)	4.2 (3.9, 4.5)	4.1 (4.0, 4.6)	4.2 (3.9, 4.4)
Plasma magnesium concentration (mmol/L)	0.96 (0.84, 1.12)	0.95 (0.84, 1.14)	1.01 (0.92, 1.16)	0.92 (0.82, 1.08)
Plasma urea concentration (mmol/L)	13.6 (8.8, 19.5)	13.8 (9.5, 20.1)	12.1 (7.8, 17.7)	13.2 (8.2, 18.5)
Plasma creatinine concentration (micromol/L)	125 (78, 214)	134 (78, 224)	108 (70, 151)	112 (84, 185)
White cell count (x109 / L)	11.1 (7.7, 16.3)	11.1 (7.5, 16.2)	10.6 (7.6, 13.4)	12.0 (9.5, 16.8)
Haemoglobin concentration (g/L)	98 (88, 113)	97 (87, 111)	103 (94, 112)	101 (90, 116)
Platelet count ($x10^9$ / L)	166 (109, 234)	163 (105, 231)	192 (118, 247)	180 (136, 237)
Prothrombin time (s)	16.1 (15.0, 19.0)	16.2 (15.0, 19.0)	15.5 (14.4, 17.0)	16.6 (15.0, 19.4)
Systolic blood pressure after AF onset	116 (99, 131)	112 (97, 128)	128 (108, 156)	119 (103, 135)
Mean blood pressure after AF onset	75 (67, 86)	74 (67, 85)	83 (73, 94)	78 (70, 88)
Heart rate after AF onset	127 (107, 147)	128 (107, 149)	125 (110, 146)	120 (98, 140)

Unadjusted time-to-event curves

Rate control

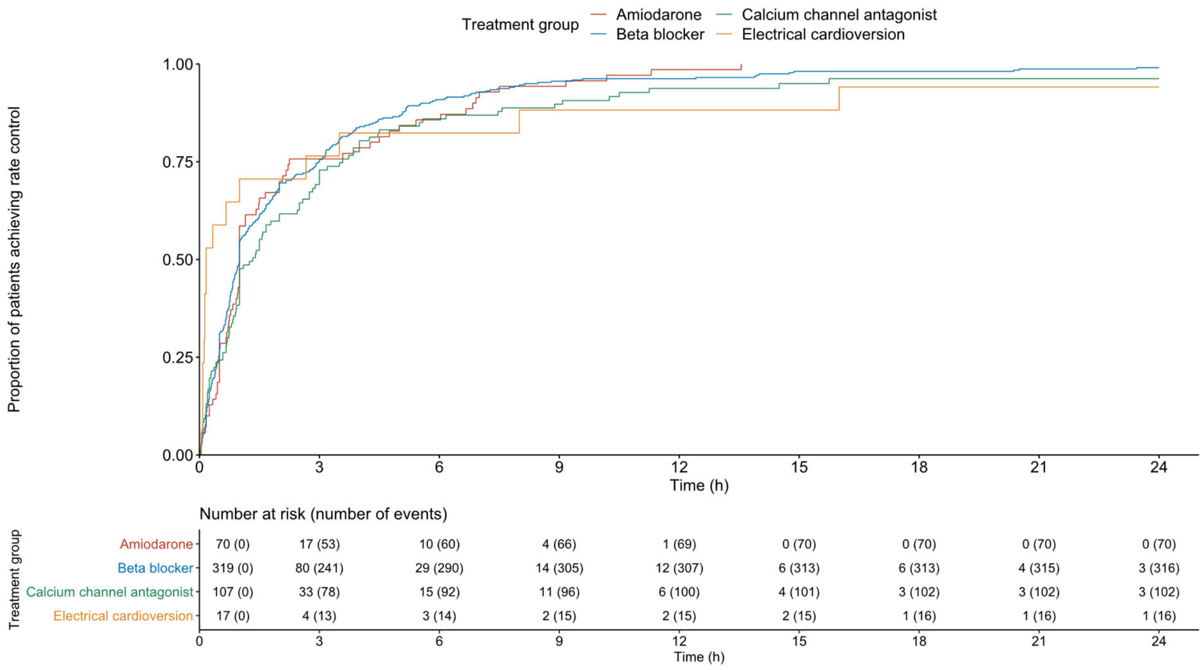

Supplemental Figure S5 - Cumulative incidence plot of time from treatment to heart rate <110 for each treatment: MIMIC database

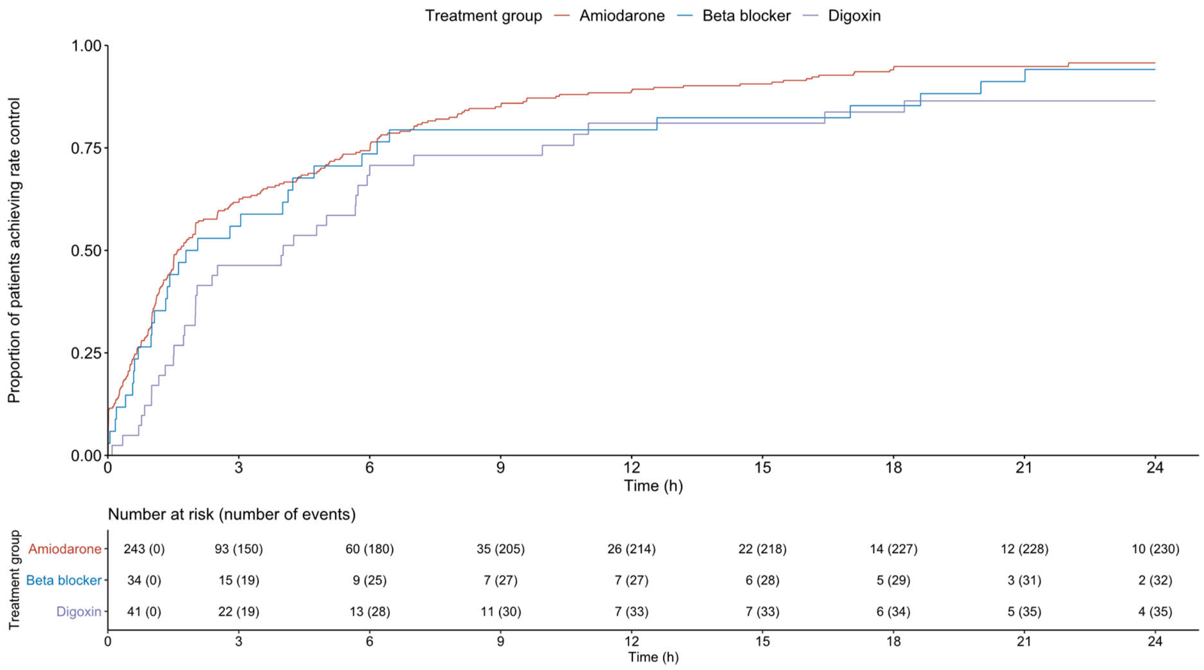

Supplemental Figure S6 - Cumulative incidence plot of time from treatment to heart rate <110 for each treatment: PICRAM database

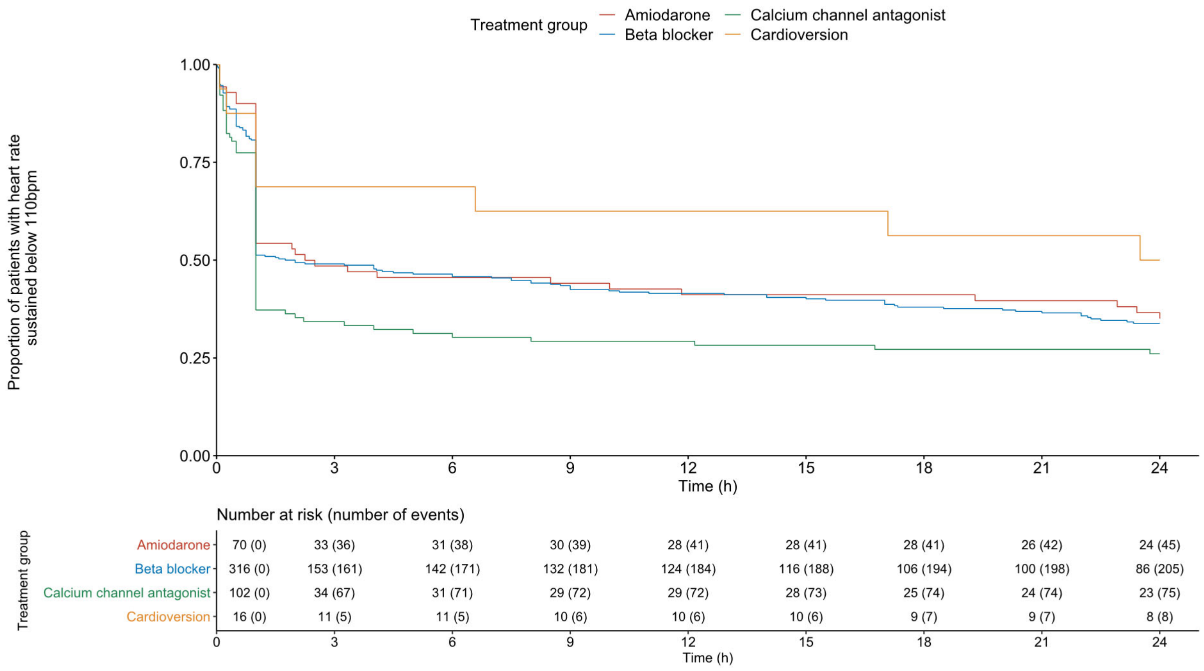

Supplemental Figure S7 - Kaplan-Meier curves of time from achieving rate control to reversion to heart rate ≥110: MIMIC-III database

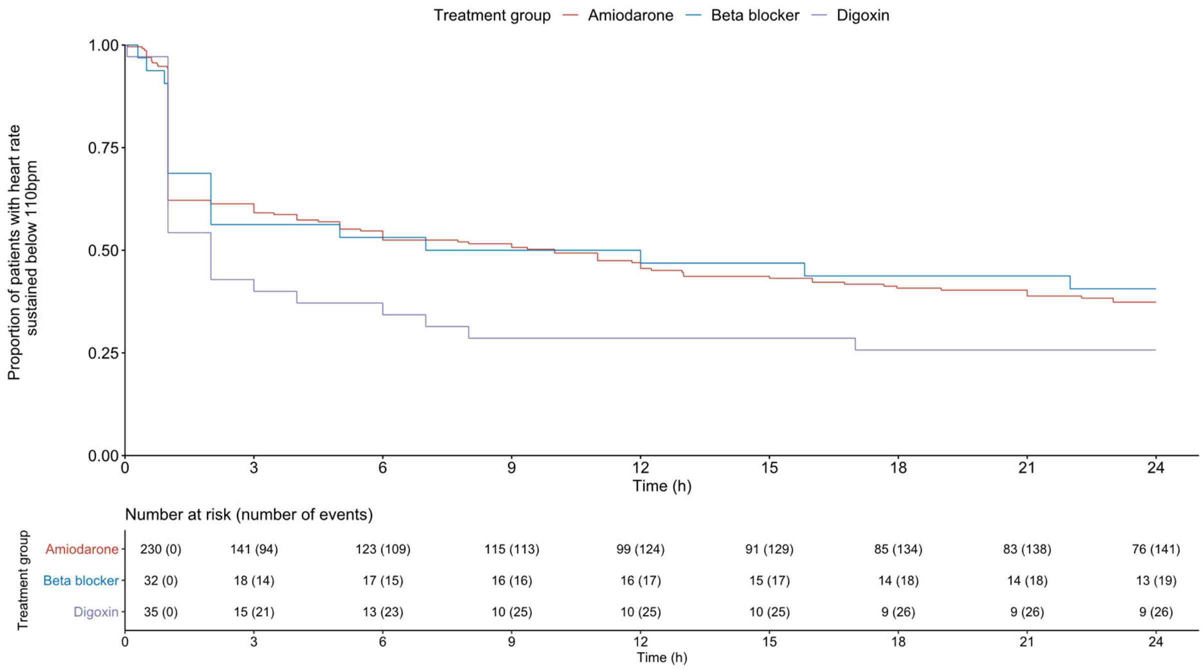

Supplemental Figure S8 - Kaplan-Meier curves of time from achieving rate control to reversion to heart rate ≥110: PICRAM database

Rhythm control

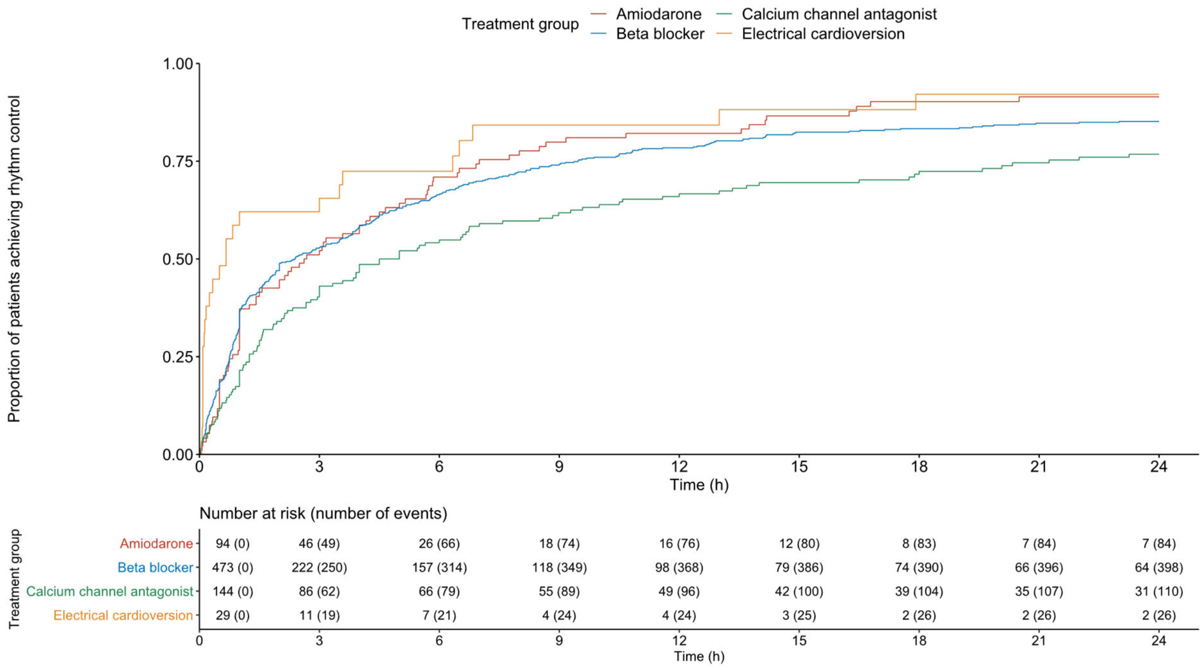

Supplemental Figure S9 - Cumulative incidence plot of time from treatment to rhythm control for each treatment: MIMIC database

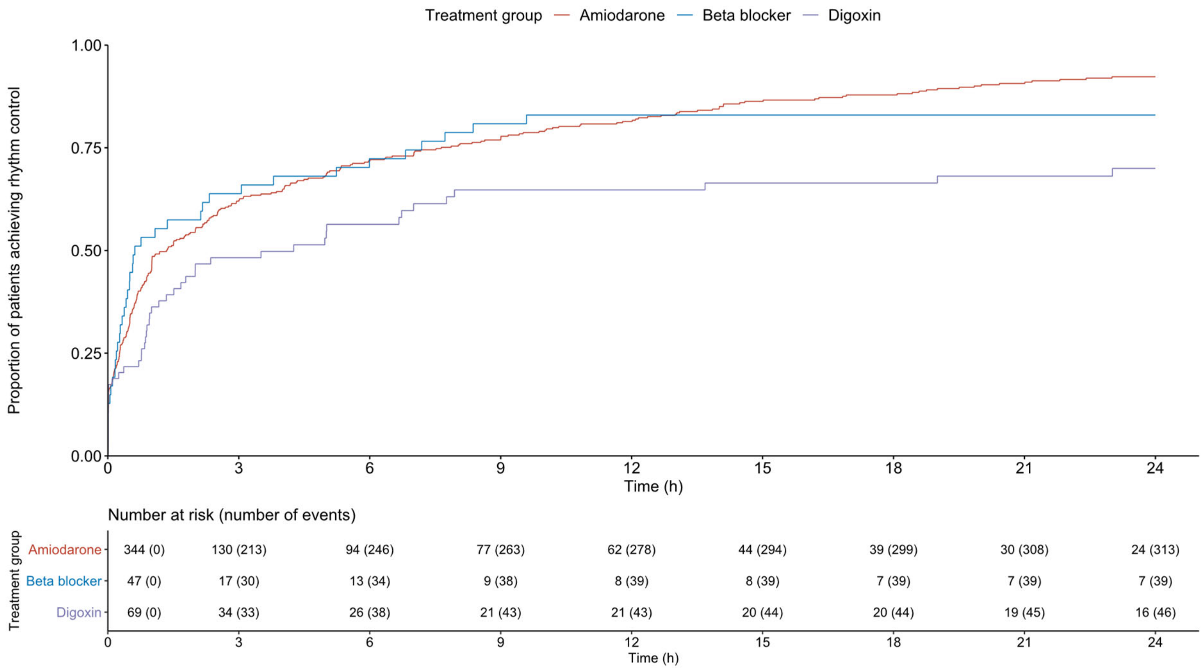

Supplemental Figure S10 - Cumulative incidence plot of time from treatment to rhythm control for each treatment: PICRAM database

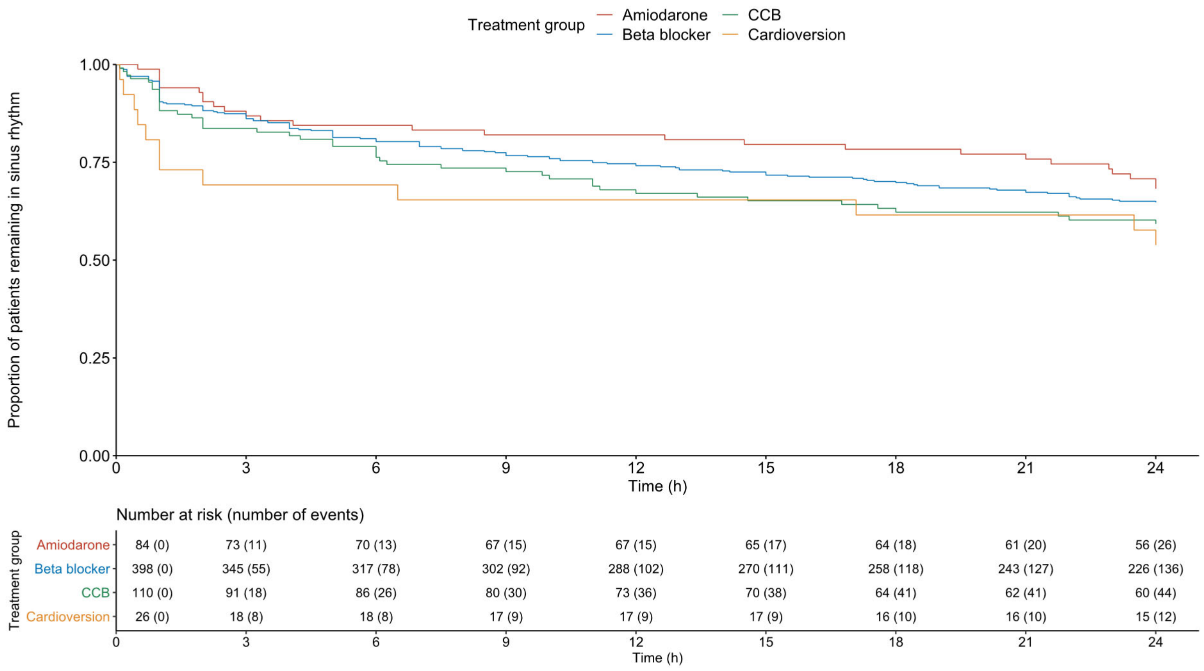

Supplemental Figure S11 - Kaplan-Meier curves for each treatment of time from achieving rhythm control to reversion to AF: MIMIC-III database

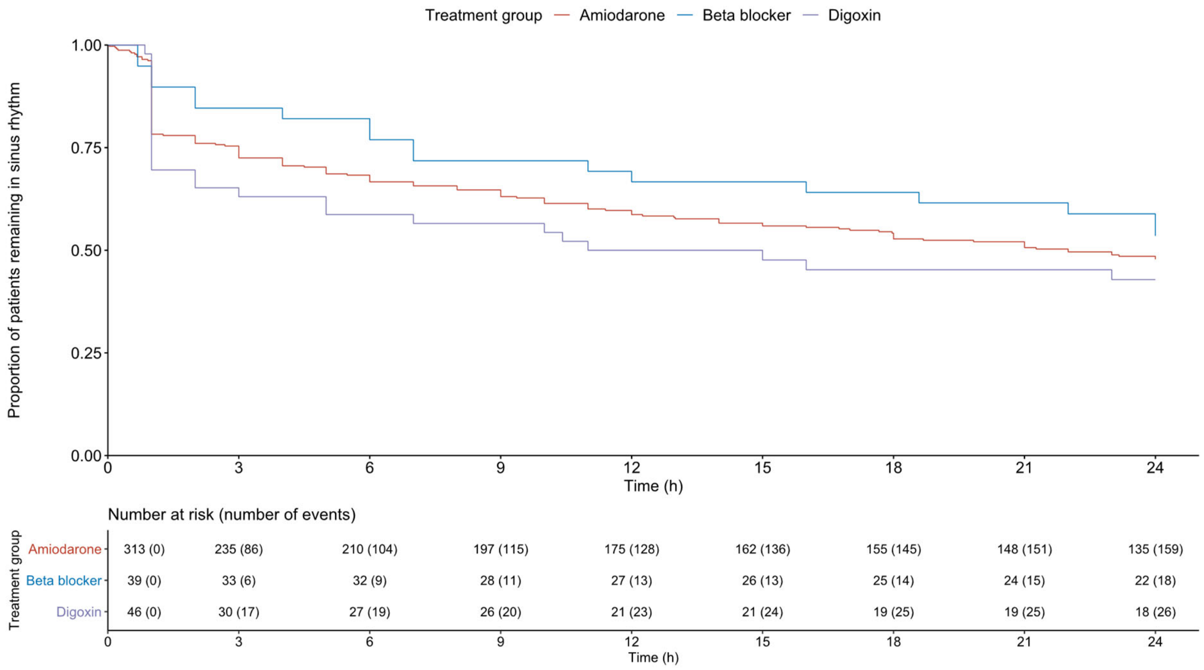

Supplemental Figure S12 - Kaplan-Meier curves for each treatment of time from achieving rhythm control to reversion to AF: PICRAM database

Survival

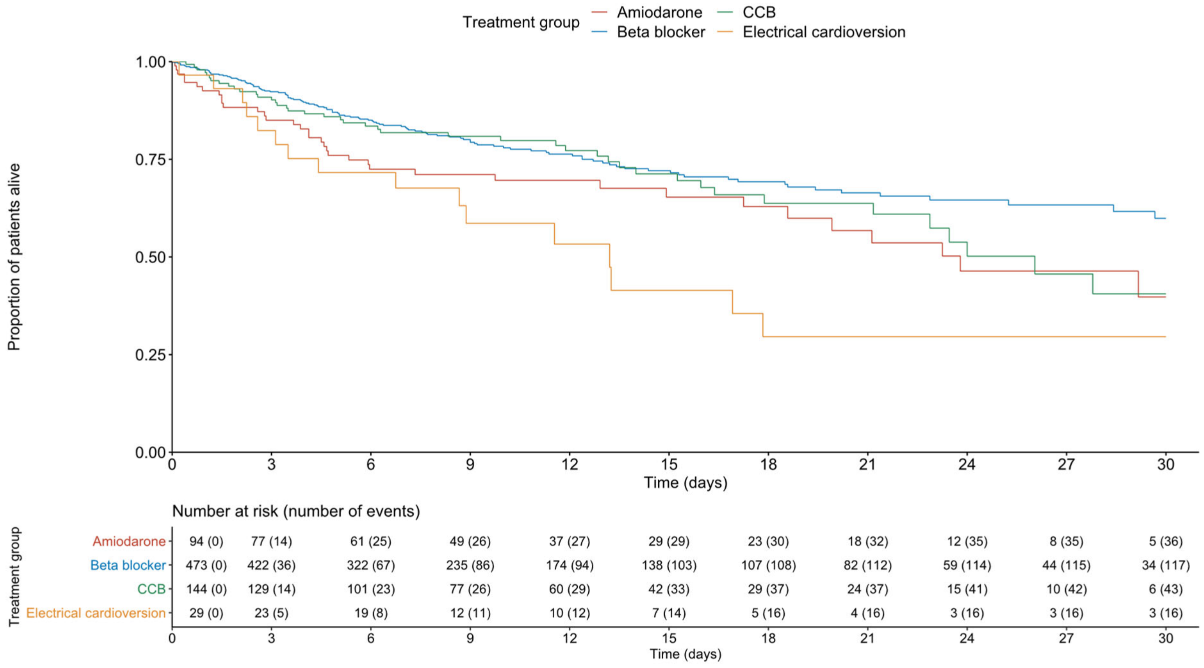

Supplemental Figure S13 - Kaplan-Meier survival curves for each treatment group: MIMIC-III database

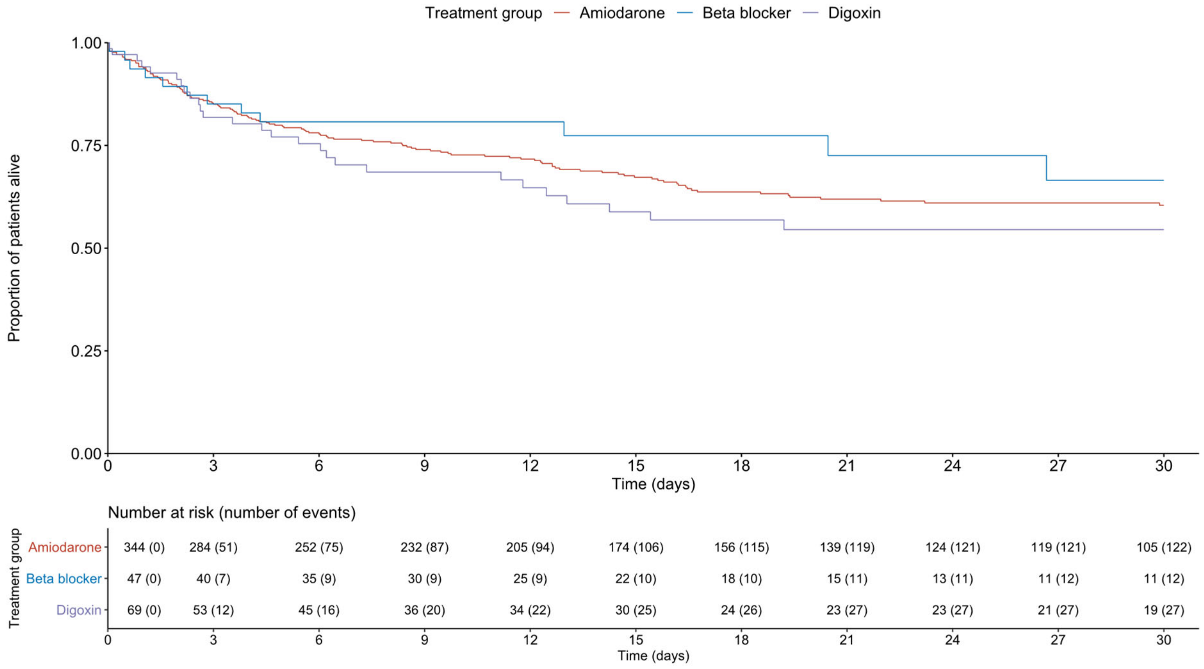

Supplemental Figure S14 - Kaplan-Meier survival curves for each treatment group: PICRAM database

Adjustment for confounding

Supplemental Table S5 - Unweighted and weighted covariate means by treatment group: MIMIC-III database

**Variable**	**Unweighted means**				**Weighted means**				**Maximum pairwise standardised mean difference (Unweighted)**	**Maximum pairwise standardised mean difference (Weighted)**
	Amiodarone	Beta blocker	Calcium channel blocker	DCCV	Amiodarone	Beta blocker	Calcium channel blocker	DCCV		
Age	71.84	71.98	72.56	74.14	72.90	72.56	72.74	72.72	0.17	0.03
Male sex	0.46	0.51	0.47	0.66	0.51	0.51	0.51	0.54	0.20	0.03
OASIS 3hr score	36.80	35.21	35.22	38.93	35.87	35.89	35.94	36.71	0.48	0.11
Beta blocker on admission	0.40	0.45	0.31	0.52	0.44	0.44	0.41	0.49	0.21	0.08
Antipsychotic medication on admission	0.05	0.06	0.03	0.00	0.04	0.04	0.04	0.00	0.06	0.04
Thyroid disorder	0.02	0.06	0.02	0.03	0.04	0.04	0.04	0.05	0.04	0.01
COPD	0.03	0.06	0.13	0.10	0.06	0.08	0.08	0.11	0.10	0.05
Liver disease	0.02	0.01	0.01	0.00	0.01	0.01	0.01	0.00	0.02	0.01
Dialysis-dependent Renal failure	0.00	0.00	0.00	0.00	0.00	0.00	0.00	0.00	0.00	0.00
Plasma sodium concentration (mmol/L)	138	140	140	139	139	140	140	140	0.35	0.03
Plasma potassium concentration (mmol/L)	4.10	4.02	4.02	4.03	4.01	4.02	4.01	3.97	0.15	0.08
Plasma magnesium concentration (mmol/L)	0.86	0.86	0.87	0.84	0.85	0.85	0.85	0.83	0.20	0.17

Plasma creatinine concentration (mmol/L)	164.2	148.1	125.4	219.5	157.1	152.4	149.9	163.6	0.57	0.08
Plasma urea concentration (micromol/L)	12.0	12.00	12.1	18.5	12.3	13.0	13.3	15.1	0.69	0.30
White cell count	14.9	12.7	12.6	15.3	13.5	13.3	12.9	13.8	0.32	0.11
Haemoglobin concentration (g/L)	103	104	105	100	103	103	103	101	0.30	0.13
Platelet count (x10^9 / L)	207	215	221	184	206	209	209	200	0.29	0.07
Therapeutic anticoagulation at time of NOAF	0.06	0.05	0.03	0.07	0.05	0.04	0.04	0.04	0.03	0.01
Prothrombin time	15.8	14.9	14.9	15.5	15.2	15.2	15.2	15.5	0.24	0.10
Systolic blood pressure after AF onset	106	119	117	90	113	114	114	109	0.83	0.15
Mean blood pressure after AF onset	67	75	74	58	71	71	72	68	0.76	0.20
Heart rate after AF onset	124	121	124	122	123	123	123	125	0.11	0.09
Temp (°C)	37.0	37.0	37.0	36.9	37.0	37.0	37.1	37.0	0.23	0.05
IV Vasoactive medication at time of NOAF	0.36	0.10	0.06	0.45	0.17	0.16	0.15	0.21	0.39	0.06
Noradrenaline dose (mcg/Kg/min)	0.12	0.02	0.01	0.08	0.03	0.03	0.02	0.04	0.66	0.09
Vasopressin dose (mcg/Kg/min)	0.17	0.05	0.02	0.29	0.07	0.07	0.05	0.10	0.48	0.08
Bronchodilator therapy on day of,	0.35	0.33	0.43	0.28	0.33	0.33	0.35	0.30	0.15	0.04

or day preceding, NOAF										
Mechanical ventilation at time of NOAF	0.64	0.44	0.38	0.76	0.51	0.50	0.47	0.58	0.38	0.11
Central venous catheter at time of NOAF	0.73	0.55	0.52	0.83	0.63	0.61	0.61	0.68	0.31	0.08
Renal replacement therapy during or <12h prior to NOAF	0.05	0.08	0.01	0.10	0.04	0.05	0.02	0.04	0.10	0.03

Supplemental Table S6 - Unweighted and weighted covariate means by treatment group: PICRAM database

**Variable**	**Unweighted means**			**Weighted means**			**Maximum pairwise standardised mean difference (Unweighted)**	**Maximum pairwise standardised mean difference (Weighted)**
	Amiodarone	Beta blocker	Digoxin	Amiodarone	Beta blocker	Digoxin		
Age	68.60	68.94	71.78	69.52	69.23	70.24	0.28	0.09
Male sex	0.59	0.57	0.64	0.60	0.59	0.62	0.06	0.02
OASIS 3hr score	33.77	31.17	29.83	32.75	32.62	31.91	0.43	0.09
Beta blocker on admission	0.13	0.21	0.13	0.14	0.16	0.15	0.08	0.02
Antipsychotic medication on admission	0.01	0.02	0.01	0.02	0.02	0.01	0.01	0.01
Thyroid disorder	0.06	0.06	0.06	0.06	0.06	0.07	0.01	0.00
COPD	0.15	0.02	0.16	0.12	0.05	0.13	0.14	0.07
Liver disease	0.04	0.02	0.07	0.04	0.03	0.05	0.05	0.02
NYHA class III/IV heart failure	0.01	NA	0.00	0.00	NA	0.00	0.01	0.00

Dialysis-dependent renal failure	0.02	0.00	0.01	0.01	0.00	0.01	0.02	0.01
Plasma sodium concentration (mmol/L)	138	140	138	138	139	138	0.40	0.14
Plasma potassium concentration (mmol/L)	4.24	4.30	4.20	4.24	4.26	4.24	0.19	0.05
Plasma magnesium concentration (mmol/L)	0.99	1.02	0.96	0.99	1.00	0.99	0.29	0.08
Plasma creatinine concentration (mmol/L)	171	142	156	165	165	165	0.25	0.00
Plasma urea concentration (micromol/L)	16.0	13.7	13.9	15.0	14.6	14.3	0.27	0.09
White cell count	12.6	11.2	12.9	12.5	11.9	12.5	0.25	0.09
Haemoglobin concentration (g/L)	101	104	105	102	102	102	0.27	0.03
Platelet count ($x10^9$ / L)	180	198	208	190	197	197	0.25	0.06
Therapeutic anticoagulation at time of NOAF	0.11	0.11	0.09	0.11	0.13	0.11	0.02	0.02
Prothrombin time	17.5	16.4	18.5	17.5	16.8	17.8	0.41	0.18
Systolic blood pressure after AF onset	115	131	121	118	122	118	0.64	0.13
Mean blood pressure after AF onset	77	85	81	79	79	79	0.48	0.04
Heart rate after AF onset	129	127	120	127	126	127	0.31	0.03
Temp (°C)	36.6	36.8	36.5	36.6	36.8	36.8	0.34	0.17
IV Vasoactive medication at time of NOAF	0.31	0.13	0.19	0.26	0.22	0.24	0.18	0.04
Noradrenaline dose (mcg/Kg/min)	0.06	0.04	0.04	0.06	0.05	0.06	0.23	0.05
Vasopressin dose (mcg/Kg/min)	0.05	NA	0.00	0.02	NA	0.00	0.29	0.12

Bronchodilator therapy on day of, or day preceding, NOAF	0.17	0.15	0.16	0.17	0.18	0.16	0.02	0.02
Mechanical ventilation at time of NOAF	0.56	0.47	0.42	0.52	0.53	0.49	0.14	0.04
Central venous catheter at time of NOAF	0.76	0.68	0.46	0.69	0.71	0.64	0.30	0.07
Renal replacement therapy during or <12h prior to NOAF	0.15	0.11	0.12	0.14	0.14	0.13	0.04	0.01

Supplemental Table S7 - Unweighted and weighted covariate means by treatment group: Combined databases

**Variable**	**Unweighted means**		**Weighted means**		**Pairwise standardised mean difference (Unweighted)**	**Pairwise standardised mean difference (Weighted)**
	Amiodarone	Beta blockers	Amiodarone	Beta blockers		
Age	69.30	71.70	70.65	70.54	0.20	-0.01
Male sex	0.56	0.51	0.54	0.53	-0.05	0.00
OASIS 3hr score	34.42	34.84	34.65	34.66	0.05	0.00
Beta blocker on admission	0.19	0.44	0.32	0.34	0.25	0.02
Antipsychotic medication on admission	0.02	0.04	0.03	0.03	0.02	0.00
Thyroid disorder	0.05	0.06	0.05	0.06	0.01	0.00
COPD	0.12	0.06	0.09	0.08	-0.07	0.00
Liver disease	0.04	0.02	0.02	0.02	-0.02	0.00
NYHA class III/IV heart failure	0.00	0.00	0.00	0.00	0.00	0.00
Dialysis-dependent renal failure	0.01	0.00	0.01	0.01	-0.01	0.00
Plasma sodium concentration (mmol/L)	138	140	139	139	0.35	0.01
Plasma potassium concentration (mmol/L)	4.21	4.05	4.12	4.12	-0.29	-0.01
Plasma magnesium concentration (mmol/L)	0.96	0.87	0.92	0.91	-0.43	-0.02
Plasma creatinine concentration (mmol/L)	174	147	157	159	-0.17	0.01
Plasma urea concentration (micromol/L)	15.3	12.1	13.7	13.5	-0.32	-0.02
White cell count	13.2	12.5	12.8	13.0	-0.09	0.01
Haemoglobin concentration (g/L)	101	104	103	103	0.17	0.01
Platelet count ($x10^9$ / L)	186	213	200	201	0.22	0.01
Therapeutic anticoagulation at time of NOAF	0.10	0.05	0.08	0.07	-0.04	0.00

Prothrombin time	16.9	15.0	16.0	16.0	-0.45	-0.01
Systolic blood pressure after AF onset	113	124	119	119	0.39	0.01
Mean blood pressure after AF onset	76	82.	79	79	0.33	0.03
Heart rate after AF onset	128	120	123	123	-0.29	0.00
Temp (°C)	36.68	37.00	36.86	36.87	0.37	0.02
IV Vasoactive medication at time of NOAF	0.32	0.10	0.20	0.19	-0.22	-0.01
Noradrenaline dose (mcg/Kg/min)	0.08	0.02	0.04	0.04	-0.43	-0.02
Vasopressin dose (mcg/Kg/min)	0.07	0.04	0.06	0.07	-0.09	0.02
Bronchodilator therapy on day of, or day preceding, NOAF	0.21	0.31	0.26	0.27	0.11	0.00
Mechanical ventilation at time of NOAF	0.58	0.44	0.50	0.50	-0.14	0.00
Central venous catheter at time of NOAF	0.76	0.56	0.65	0.66	-0.19	0.00
Renal replacement therapy during or <12h prior to NOAF	0.13	0.08	0.10	0.11	-0.05	0.00

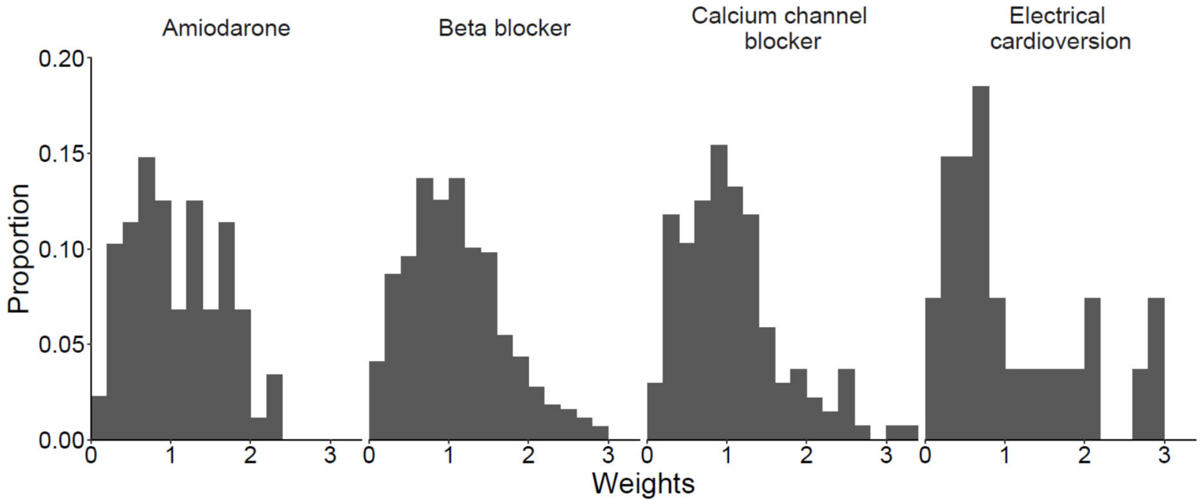

Supplemental Figure S15 - Histograms of propensity weights by treatment group: MIMIC-III database

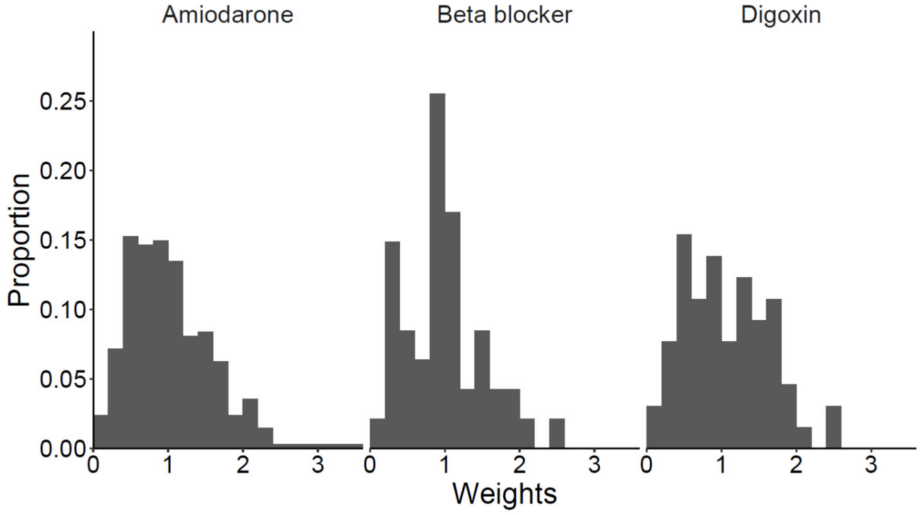

Supplemental Figure S16 - Histograms of propensity weights by treatment group: PICRAM database

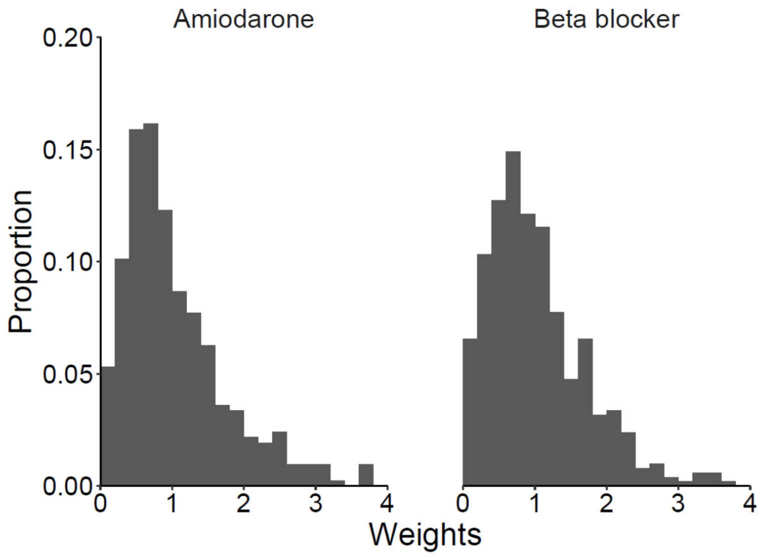

Supplemental Figure S17 - Histograms of propensity weights by treatment group: Combined dataset

Outcomes

Supplemental Table S8 - Unadjusted and adjusted hazard ratios for each outcome (compared with amiodarone) and associated 95% confidence intervals: MIMIC-III database

**Outcome**	**Treatment group**	**Unadjusted**			**Adjusted**		
		Hazard ratio	%95CI Lower	95% CI Upper	Hazard ratio	%95CI Lower	95% CI Upper
**Rate control**	Beta blocker	1.03	0.81	1.30	1.09	0.78	1.51
	Calcium channel blocker	0.83	0.62	1.12	0.81	0.55	1.19
	Cardioversion	1.01	0.39	2.62	1.59	0.44	5.75
**Rhythm control**	Beta blocker	0.91	0.73	1.12	0.91	0.61	1.35
	Calcium channel blocker	0.65	0.50	0.84	0.59	0.37	0.92
	Cardioversion	1.45	0.82	2.57	2.00	0.86	4.65
**Reversion to AF**	Beta blocker	1.18	0.79	1.78	1.37	0.67	2.78
	Calcium channel blocker	1.44	0.90	2.31	1.73	0.78	3.84
	Cardioversion	1.80	0.83	3.90	1.01	0.28	3.71
**Reversion to heart rate ≥110**	Beta blocker	1.08	0.80	1.46	0.95	0.59	1.52
	Calcium channel blocker	1.44	1.00	2.07	1.61	0.93	2.79
	Cardioversion	0.67	0.30	1.53	0.93	0.36	2.42
**Hospital mortality**	Beta blocker	0.64	0.44	0.93	1.03	0.53	2.03
	Calcium channel blocker	0.77	0.50	1.20	1.30	0.61	2.76
	Cardioversion	1.56	0.86	2.83	0.96	0.31	3.01

Supplemental Table S9 - Unadjusted and adjusted hazard ratios (compared with amiodarone) for each outcome and associated 95% confidence intervals: PICRAM database

**Outcome**	**Treatment group**	**Unadjusted results**			**Adjusted results**		
		Hazard ratio	%95CI Lower	95% CI Upper	Hazard ratio	%95CI Lower	95% CI Upper
**Rate control**	Beta blocker	0.85	0.57	1.27	0.82	0.48	1.42
	Digoxin	0.64	0.45	0.92	0.56	0.34	0.92
**Rhythm control**	Beta blocker	0.95	0.64	1.40	0.99	0.57	1.72
	Digoxin	0.57	0.41	0.81	0.67	0.41	1.09
**Reversion to AF**	Beta blocker	0.79	0.50	1.27	0.84	0.42	1.65
	Digoxin	1.21	0.78	1.89	1.32	0.71	2.47
**Reversion to HR ≥110**	Beta blocker	0.94	0.58	1.52	0.88	0.43	1.79
	Digoxin	1.41	0.91	2.19	1.14	0.63	2.09
**Hospital mortality**	Beta blocker	0.74	0.40	1.38	0.75	0.30	1.86
	Digoxin	1.21	0.79	1.86	1.40	0.77	2.56

Supplemental Table S10 - Unadjusted and adjusted hazard ratios for each outcome (compared with amiodarone) and associated 95% confidence intervals: Combined databases

**Outcome**	**Treatment group**	**Unadjusted results**			**Adjusted results**		
		Hazard ratio	%95CI Lower	95% CI Upper	Hazard ratio	%95CI Lower	95% CI Upper
**Rate control**	Beta blocker	1.32	1.12	1.55	1.15	0.91	1.46
**Rhythm control**	Beta blocker	0.81	0.71	0.93	0.85	0.69	1.05
**Reversion to AF**	Beta blocker	0.68	0.55	0.85	0.75	0.54	1.03
**Reversion to heart rate ≥110**	Beta blocker	1.16	0.95	1.42	1.02	0.76	1.37
**Hospital mortality**	Beta blocker	0.77	0.61	0.98	1.06	0.73	1.52

Missingness of variables

Supplemental Table S11 - Missingness of weighting variables: MIMIC-III database

Variable	Number missing	Percentage missing
Prothrombin time	194	26.22
Beta blocker on admission	72	9.73
Antipsychotic medication on admission	72	9.73
Plasma magnesium concentration (mmol/L)	26	3.51
White cell count	11	1.49
Platelet count (x10^9 / L)	11	1.49
Haemoglobin concentration (g/L)	10	1.35
Plasma creatinine concentration (mmol/L)	9	1.22
Plasma urea concentration (micromol/L)	9	1.22
Plasma sodium concentration (mmol/L)	6	0.81
Plasma potassium concentration (mmol/L)	4	0.54

Supplemental Table S12 - Missingness of matching variables: PICRAM database

Variable	Number missing	Percentage missing
Prothrombin time	49	10.65
White cell count	44	9.57
Plasma creatinine concentration (mmol/L)	41	8.91
Platelet count (x10^9 / L)	39	8.48
Plasma magnesium concentration (mmol/L)	35	7.61
Plasma urea concentration (micromol/L)	35	7.61
Haemoglobin concentration (g/L)	3	0.65
Plasma sodium concentration (mmol/L)	2	0.43
potassium	2	0.43
Mean blood pressure after AF onset	1	0.22
Systolic blood pressure after AF onset	1	0.22
