# Supplementary material for: Comparative effectiveness of common treatments for new-onset atrial fibrillation within the ICU: Accounting for physiological status
Source: J Crit Care. 2022 Feb;67:149–56. doi: 10.1016/j.jcrc.2021.11.005 (PMC8687206; doi:10.1016/j.jcrc.2021.11.005)
Supplement: Supplementary file 3 — Supplementary material 3 [file mmc3.pdf]

Additional File 3

Sensitivity analyses

1 AMIODARONE VS BETA BLOCKERS

Supplemental Table S1 - Unweighted and weighted covariate means by treatment group: MIMIC-III database

	Amiodarone unweighted	Beta blocker unweighted	Amiodarone weighted	Beta blocker weighted	Standardised mean difference (Unweighted)	Standardised mean difference (weighted)
Age	71.8	72.0	72.4	72.0	0.010	-0.022
Male sex	0.46	0.51	0.50	0.50	0.048	0.002
OASIS 3hr score	36.8	35.2	35.6	35.5	-0.204	-0.009
Beta blocker on admission	0.47	0.44	0.46	0.45	-0.022	-0.008
Antipsychotic medication on admission (%)	5	5	5	5	-0.005	-0.001
Thyroid disorder (%)	2	6	4	5	0.036	0.011
COPD (%)	3	6	4	5	0.027	0.010
Liver disease (%)	2	2	1	2	-0.006	0.002
Dialysis-dependent Renal failure (%)	0	0.2	0	0.2	0.002	0.002
Plasma sodium concentration (mmol/L)	138	140	139	139	0.270	0.034
Plasma potassium concentration (mmol/L)	4.10	4.02	4.0	4.0	-0.131	0.005
Plasma magnesium concentration (mmol/L)	0.85	0.86	0.86	0.86	0.032	-0.002

Plasma creatinine concentration (mmol/L)	165	147	153	151	-0.104	-0.016
Plasma urea concentration (micromol/L)	12.0	11.9	11.6	11.9	-0.011	0.039
White cell count	14.9	12.7	13.4	13.2	-0.279	-0.023
Haemoglobin concentration (g/L)	103	104	104	104	0.053	0.004
Platelet count ($x10^9$ / L)	207	214	210	212	0.052	0.012
Therapeutic anticoagulation at time of NOAF (%)	6	5	6	5	-0.015	-0.005
Prothrombin time (s)	15.9	15.1	15.1	15.2	-0.200	0.019
Systolic blood pressure after AF onset	106	119	116	117	0.388	0.021
Mean blood pressure after AF onset	67	75	73	73	0.350	0.016
Heart rate after AF onset	124	121	122	121	-0.118	-0.026
Temp (°C)	37.0	37.0	37.0	37.0	-0.028	-0.004
IV Vasoactive medication at time of NOAF	0.36	0.1	0.158	0.143	-0.267	-0.015
Noradrenaline dose (mcg/Kg/min)	0.115	0.016	0.036	0.028	-0.493	-0.042
Vasopressin dose (mcg/Kg/min)	0.167	0.048	0.068	0.070	-0.244	0.005
Bronchodilator therapy on day of, or day preceding, NOAF	35.1	32.8	33.4	32.8	-0.023	-0.006
Mechanical ventilation at time of NOAF (%)	64	44	49	47	-0.203	-0.020
Central venous catheter at time of NOAF (%)	73	55	60	59	-0.182	-0.014
Renal replacement therapy during or <12h prior to NOAF (%)	5	8	5	7	0.027	0.020

Supplemental Table S2 - Adjusted hazard ratios for beta blockers (versus amiodarone)

Outcome	Hazard ratio
Hospital mortality	1.09 [0.63 - 1.89]
Rate control	1.04 [0.75 - 1.46]
Rhythm control	0.90 [0.66 - 1.23]
AF reversion	1.55 [0.86 - 2.81]
Rate reversion	1.10 [0.7 - 1.72]

2 AMIODARONE VS CALCIUM CHANNEL BLOCKERS

Supplemental Table S3 - Unweighted and weighted covariate means by treatment group: MIMIC-III database

	Amiodarone unweighted	CCB unweighted	Amiodarone weighted	CCB weighted	Standardised mean difference (Unweighted)	Standardised mean difference (weighted)
Age	71.844	72.558	72.635	72.619	0.054	-0.001
Male sex (%)	45.7	46.5	46.5	47.2	0.008	0.007
OASIS 3hr score	36.798	35.222	35.999	35.904	-0.197	-0.012
Beta blocker on admission	46.8	31.2	39.9	37.4	-0.156	-0.026
Antipsychotic medication on admission	5.3	3.5	4.8	4.5	-0.018	-0.002
Thyroid disorder	2.1	2.1	2.2	2.6	0.000	0.005
COPD	3.2	13.2	5.8	8.7	0.100	0.029
Liver disease	2.1	1.4	1.2	1.7	-0.007	0.005
Plasma sodium concentration (mmol/L)	138	140	139	139	0.349	0.042
Plasma potassium concentration (mmol/L)	4.1	4.0	4.0	4.0	-0.138	-0.002
Plasma magnesium concentration (mmol/L)	0.85	0.87	0.86	0.86	0.108	0.000
Plasma creatinine concentration (mmol/L)	165	124	145	140	-0.279	-0.037
Plasma urea concentration (micromol/L)	12.0	12.1	11.7	12.5	0.008	0.091
White cell count	14.9	12.6	13.8	13.1	-0.297	-0.087
Haemoglobin concentration (g/L)	103	105	104	104	0.104	0.008
Platelet count (x10^9 / L)	207	220	213	212	0.092	-0.005
Therapeutic anticoagulation at time of NOAF	0.064	0.035	0.049	0.043	-0.029	-0.006

Prothrombin time	15.9	15.1	15.3	15.4	-0.222	0.008
Systolic blood pressure after AF onset (mmHg)	106	117	113	113	0.315	-0.005
Mean blood pressure after AF onset (mmHg)	67	74	71	72	0.332	0.049
Heart rate after AF onset	124	124	124	124	-0.005	0.002
Temp (°C)	37.0	37.1	37.0	37.1	0.026	0.025
IV Vasoactive medication at time of NOAF	0.36	0.06	0.19	0.16	-0.299	-0.032
Noradrenaline dose (mcg/Kg/min)	0.115	0.008	0.046	0.022	-0.541	-0.120
Vasopressin dose (mcg/Kg/min)	0.167	0.017	0.071	0.055	-0.336	-0.035
Bronchodilator therapy on day of, or day preceding, NOAF	35	43	38	39	0.079	0.012
Mechanical ventilation at time of NOAF	64	38	50	46	-0.256	-0.037
Central venous catheter at time of NOAF	73	52	62	60	-0.213	-0.020
Renal replacement therapy during or <12h prior to NOAF	5	1	2	2	-0.046	-0.005

Supplemental Table S4 - Adjusted hazard ratios for calcium channel blockers (versus amiodarone)

Hospital mortality	1.32 [0.7 - 2.49]
Rate control	0.73 [0.49 - 1.09]
**Rhythm control**	**0.55 [0.37 - 0.82]**
AF reversion	1.79 [0.91 - 3.53]
**Rate reversion**	**1.82 [1.10 – 3.00]**

3 AMIODARONE VS ELECTRICAL CARDIOVERSION

Supplemental Table S5 - Unweighted and weighted covariate means by treatment group: MIMIC-III database

	Amiodarone unweighted	Cardioversion unweighted	Amiodarone weighted	Cardioversion weighted	pairwise standardised mean difference (Unweighted)	pairwise standardised mean difference (weighted)
Age	71.8	74.1	72.3	72.2	0.054	-0.001
Male sex (%)	45.7	65.5	52.4	56.3	0.008	0.007
OASIS 3hr score	3679.8	3893.1	3711.7	3750.4	-0.197	-0.012
Beta blocker on admission (%)	46.8	55.2	49	50.8	-0.156	-0.026
Antipsychotic medication on admission (%)	5.3	3.4	4.2	3.8	-0.018	-0.002
Thyroid disorder (%)	2.1	3.4	2.5	3.7	0.000	0.005
COPD (%)	3.2	10.3	4.8	8.1	0.100	0.029
Liver disease (%)	2.1	0	1.4	0	-0.007	0.005
Plasma sodium concentration (mmol/L)	138	139	138	139	0.349	0.042
Plasma potassium concentration (mmol/L)	4.1	4	4.1	4	-0.138	-0.002
Plasma magnesium concentration (mmol/L)	0.85	0.84	0.85	0.84	0.108	0.000
Plasma creatinine concentration (mmol/L)	165	222	182	187	-0.279	-0.037
Plasma urea concentration (micromol/L)	12	18.9	13.6	16.4	0.008	0.091
White cell count	14.9	15.3	14.9	14.6	-0.297	-0.087
Haemoglobin concentration (g/L)	103	100	101	101	0.104	0.008
Platelet count ($x10^9$ / L)	207	182	196	190	0.092	-0.005

Therapeutic anticoagulation at time of NOAF	6.4	6.9	6	5.2	-0.029	-0.006
Prothrombin time (s)	15.9	16.1	16.1	16.4	-0.222	0.008
Systolic blood pressure after AF onset (mmHg)	106	90	102	97	0.315	-0.005
Mean blood pressure after AF onset (mmHg)	67	58	64	62	0.332	0.049
Heart rate after AF onset	124	122	124	124	-0.005	0.002
Temp (°C)	37	36.9	37	37	0.026	0.025
IV Vasoactive medication at time of NOAF (%)	36.2	44.8	37.9	37.5	-0.299	-0.032
Noradrenaline dose (mcg/Kg/min)	0.115	0.084	0.1	0.066	-0.541	-0.120
Vasopressin dose (mcg/Kg/min)	0.167	0.29	0.216	0.257	-0.336	-0.035
Bronchodilator therapy on day of, or day preceding, NOAF (%)	35.1	27.6	32.5	29.2	0.079	0.012
Mechanical ventilation at time of NOAF (%)	63.8	75.9	65.6	67.9	-0.256	-0.037
Central venous catheter at time of NOAF (%)	73.4	82.8	76.6	77.9	-0.213	-0.020
Renal replacement therapy during or <12h prior to NOAF (%)	5.3	10.3	6.5	8	-0.046	-0.00

Supplemental Table S6 - Adjusted hazard ratios for electrical cardioversion (versus amiodarone)

Hospital mortality	1.08 [0.5 - 2.36]
Rate control	1.04 [0.34 - 3.19]
Rhythm control	1.70 [0.84 - 3.41]
AF reversion	1.60 [0.62 - 4.1]
Rate reversion	0.55 [0.2 - 1.56]

Sensitivity analysis – PICRAM database

4 Amiodarone vs beta blockers

Supplemental Table S7 - Unweighted and weighted covariate means by treatment group: PICRAM database

	Amiodarone unweighted	Beta blocker unweighted	Amiodarone weighted	Beta blocker weighted	pairwise standardised mean difference (Unweighted)	pairwise standardised mean difference (weighted)
Age	68.6	68.9	68.7	68.5	0.030	-0.012
Male sex (%)	59.0	57.4	58.7	58.3	-0.016	-0.004
OASIS 3hr score	33.767	31.170	33.374	33.077	-0.281	-0.032
Beta blocker on admission (%)	12.8	21.3	14.5	16.5	0.085	0.019
Antipsychotic medication on admission (%)	1.5	2.1	1.7	2.2	0.007	0.005
Thyroid disorder (%)	6.1	6.4	6.5	6.5	0.003	0.000
COPD (%)	14.8	2.1	11	5	-0.127	-0.059
Liver disease (%)	4.1	2.1	3.6	3.1	-0.019	-0.005
NYHA class III/IV heart failure (%)	0.6	0	0.4	0	-0.006	-0.004
Dialysis-dependent renal failure (%)	1.7	0	1.1	0	-0.017	-0.011
Plasma sodium concentration (mmol/L)	138	140	138	139	0.404	0.142
Plasma potassium concentration (mmol/L)	4.2	4.3	4.3	4.3	0.113	0.038
Plasma magnesium concentration (mmol/L)	0.99	1.02	1.00	1.01	0.130	0.034

Plasma creatinine concentration (mmol/L)	170	134	164	160	-0.286	-0.030
Plasma urea concentration (micromol/L)	16.0	13.3	15.4	14.5	-0.271	-0.090
White cell count	12.7	11.1	12.3	11.9	-0.226	-0.067
Haemoglobin concentration (g/L)	101	104	101	102	0.214	0.040
Platelet count (x10^9 / L)	182	192	187	193	0.090	0.057
Therapeutic anticoagulation at time of NOAF	0.11	0.11	0.12	0.14	-0.001	0.019
Prothrombin time (s)	18.3	16.2	17.6	16.5	-0.36	-0.19
Systolic blood pressure after AF onset	117	124	119	120	0.209	0.044
Mean blood pressure after AF onset	75	80	76	78	0.248	0.061
Heart rate after AF onset	125	129	126	127	0.127	0.035
Temp (°C)	36.6	36.8	36.7	36.8	0.290	0.119
IV Vasoactive medication at time of NOAF	0.31	0.13	0.27	0.23	-0.178	-0.038
Noradrenaline dose (mcg/Kg/min)	0.064	0.035	0.058	0.056	-0.232	-0.024
Vasopressin dose (mcg/Kg/min)	0.049	0.000	0.030	0.000	-0.239	-0.148
Bronchodilator therapy on day of, or day preceding, NOAF (%)	17	15	17	17	-0.017	0.006
Mechanical ventilation at time of NOAF (%)	56	47	55	55	-0.090	0.005
Central venous catheter at time of NOAF (%)	76	68	75	76	-0.081	0.008
Renal replacement therapy during or <12h prior to NOAF (%)	15	11	15	14	-0.045	-0.001

Supplemental Table S8 - Adjusted hazard ratios for beta blockers (versus amiodarone)

Hospital mortality	0.73 [0.29 - 1.8]
Rate control	0.88 [0.51 - 1.52]
Rhythm control	1.07 [0.59 - 1.94]
AF reversion	0.86 [0.42 - 1.77]
Rate reversion	0.98 [0.47 - 2.01]

5 AMIODARONE VS DIGOXIN

Supplemental Table S9 - Unweighted and weighted covariate means by treatment group: MIMIC-III database

	**Amiodarone unweighted**	**Digoxin unweighted**	**Amiodarone weighted**	**Digoxin weighted**	**pairwise standardised mean difference (Unweighted)**	**pairwise standardised mean difference (weighted)**
Age	68.6	71.8	69.5	70.0	0.28	0.05
Male sex	0.59	0.64	0.61	0.62	0.05	0.01
OASIS 3hr score	33.8	29.8	33.0	32.1	-0.43	-0.09
Beta blocker on admission (%)	13	13	13	14	0.00	0.01
Antipsychotic medication on admission (%)	1	1	1	1	0.00	0.00
Thyroid disorder (%)	6	6	6	7	0.00	0.01
COPD (%)	15	16	15	15	0.01	0.00
Liver disease (%)	4	7	5	5	0.03	0.01
NYHA class III/IV heart failure (%)	1	0	0	0	-0.01	0.00
Dialysis-dependent renal failure (%)	2	1	2	2	0.00	0.00
Plasma sodium concentration (mmol/L)	138	138	138	138	0.09	0.04
Plasma potassium concentration (mmol/L)	4.24	4.20	4.23	4.24	-0.07	0.00
Plasma magnesium concentration (mmol/L)	0.99	0.95	0.99	0.98	-0.16	-0.04

Plasma creatinine concentration (mmol/L)	170	153	166	164	-0.14	-0.01
Plasma urea concentration (micromol/L)	15.9	14.1	15.2	14.5	-0.20	-0.09
White cell count	12.5	12.4	12.5	12.3	-0.01	-0.03
Haemoglobin concentration (g/L)	100	105	101	102	0.25	0.03
Platelet count (x10^9 / L)	180	214	188	196	0.27	0.07
Therapeutic anticoagulation at time of NOAF (%)	11	9	11	11	-0.02	0.00
Prothrombin time	18.3	20.2	18.8	19.3	0.19	0.05
Systolic blood pressure after AF onset	117	120	118	119	0.07	0.02
Mean blood pressure after AF onset	75	78	76	77	0.14	0.06
Heart rate after AF onset	125	118	124	124	-0.29	0.00
Temp (°C)	36.6	36.5	36.6	36.6	-0.05	-0.01
IV Vasoactive medication at time of NOAF	0.31	0.19	0.28	0.26	-0.12	-0.02
Noradrenaline dose (mcg/Kg/min)	0.06	0.04	0.06	0.06	-0.15	0.02
Vasopressin dose (mcg/Kg/min)	0.05	0.00	0.03	0.00	-0.24	-0.14
Bronchodilator therapy on day of, or day preceding, NOAF (%)	17	16	17	16	-0.01	0.00
Mechanical ventilation at time of NOAF (%)	56	42	53	51	-0.14	-0.02
Central venous catheter at time of NOAF (%)	76	46	70	65	-0.30	-0.05
Renal replacement therapy during or <12h prior to NOAF (%)	15	12	14	13	-0.04	-0.01

Supplemental Table S10 - Adjusted hazard ratios for digoxin (versus amiodarone)

Hospital mortality	1.33 [0.77 - 2.29]
**Rate control**	**0.56 [0.36 - 0.88]**
Rhythm control	0.66 [0.42 - 1.03]
AF reversion	1.31 [0.74 - 2.31]
Rate reversion	1.13 [0.65 - 1.96]

Array sensitivity analyses

6 INFERIOR RATE CONTROL ASSOCIATED WITH DIGOXIN THERAPY

Supplemental Table S11 - Examples of minimum required confounder-outcome association and confounder prevalence combinations required for an unmeasured confounder to bring the observed effect estimate to the null

Association of confounder with outcome (hazard ratio)	Prevalence of confounder in digoxin group	Prevalence of confounder in amiodarone group
0.15	0.70	0.3
0.20	0.75	0.3
0.25	0.80	0.3
0.30	0.85	0.3
0.35	0.90	0.3
0.40	0.95	0.3
0.45	1.00	0.3

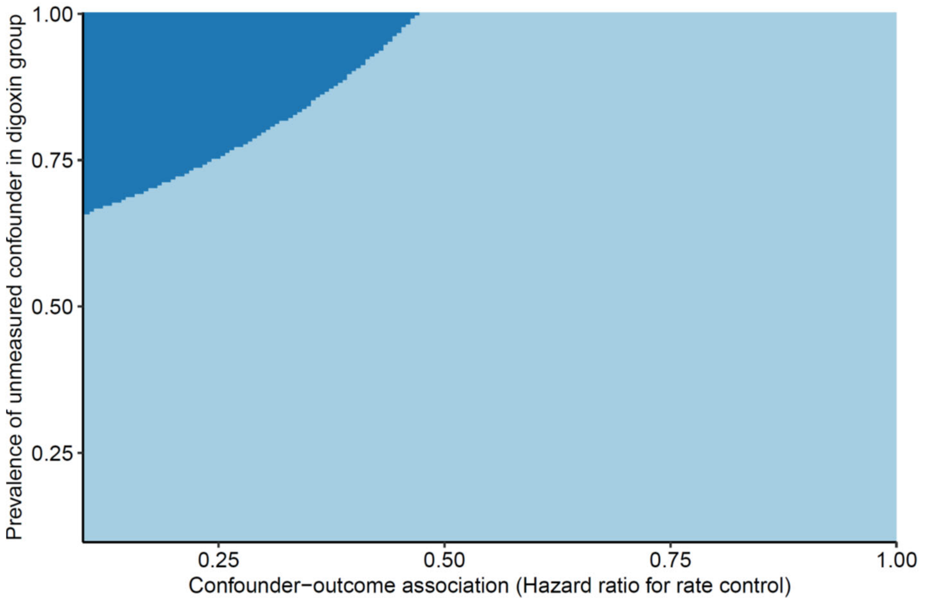

Supplemental Figure 1 - Graphical representation of confounder-outcome association and confounder prevalence combinations required to bring the observed effect estimate to the null assuming a prevalence of the unmeasured confounder of 0.3 in the reference (amiodarone) group. Dark blue represents combinations where the observed effect estimate would be brought to the null by an unmeasured confounder.

7 INFERIOR RHYTHM CONTROL ASSOCIATED WITH CCB THERAPY

Supplemental Table S12 - Examples of minimum required confounder-outcome association and confounder prevalence combinations required for an unmeasured confounder to bring the observed effect estimate to the null assuming a prevalence of the unmeasured confounder of 0.3 in the reference (amiodarone) group.

Association of confounder with outcome (hazard ratio)	Prevalence of confounder in CCB group	Prevalence of confounder in amiodarone group
0.10	0.65	0.3
0.15	0.70	0.3
0.20	0.70	0.3
0.25	0.75	0.3
0.30	0.80	0.3
0.35	0.85	0.3
0.40	0.90	0.3
0.45	0.95	0.3
0.50	1.00	0.3

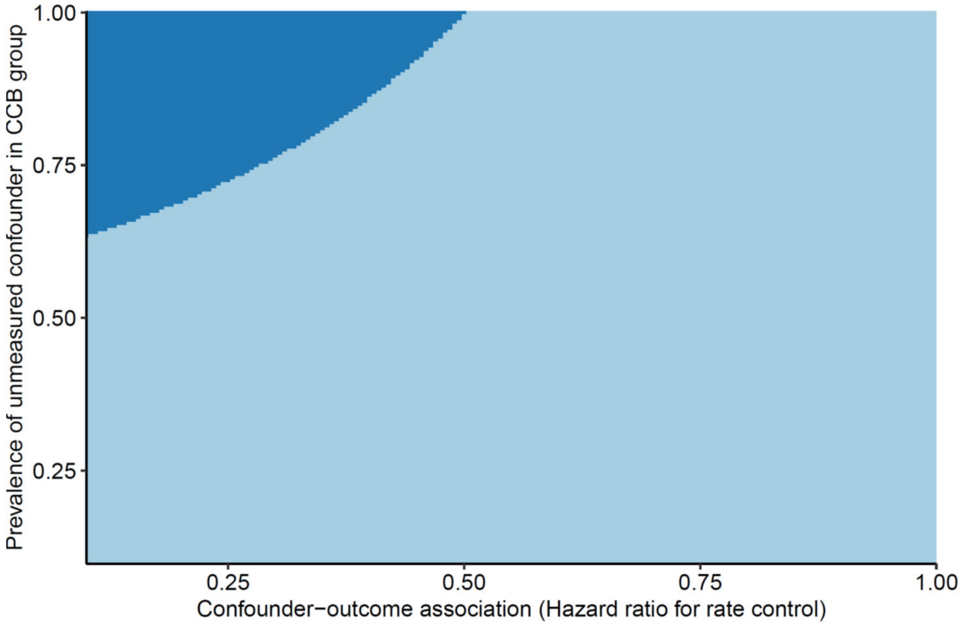

Supplemental Figure 2 - Graphical representation of confounder-outcome association and confounder prevalence combinations required to bring the observed effect estimate to the null assuming a prevalence of the unmeasured confounder of 0.3 in the reference (amiodarone) group. Dark blue represents combinations where the observed effect estimate would be brought to the null by an unmeasured confounder.
